# L-Selenomethylselenocysteine Improves Growth Performance, Antioxidant Status, and Meat Quality in White-Feathered Broilers More Effectively than Selenomethionine

**DOI:** 10.3390/ani16182882

**Published:** 2026-09-13

**Authors:** Xue Yang, Xuebing Huang, Weiguang Xia, Kaichao Li, Yanan Zhang, Shuang Wang, Wei Chen, Shenglin Wang, Chuntian Zheng

**Affiliations:** 1 State Key Laboratory of Swine and Poultry Breeding Industry, Key Laboratory of Animal Nutrition and Feed Science in South China, Ministry of Agriculture and Rural Affairs, Guangdong Provincial Key Laboratory of Animal Breeding and Nutrition, Institute of Animal Science, Guangdong Academy of Agricultural Sciences, Guangzhou 510640, China; snow_yang2017@163.com (X.Y.);; 2 Jiangxi Province Key Innovation Center of High-Quality and Safe Livestock Production, Jiangxi Province Key Laboratory of Animal Nutrition, Engineering Research Center of Feed Development, Jiangxi Agricultural University, Nanchang 330045, China

**Keywords:** L-selenomethylselenocysteine, selenomethionine, broilers, antioxidant capacity, meat quality, relative biological potency

## Abstract

Selenium (Se) is essential in poultry nutrition for antioxidant defense through selenoproteins. While selenomethionine (Se-Met) is commonly used, L-selenomethylselenocysteine (L-SeMC) may offer more favorable responses for selected measured variables. This study compared Se-Met and L-SeMC effects on growth, antioxidant status, and meat quality in broilers. Six hundred and sixty one-day-old male Cobb 500 broiler chickens were randomly assigned to 11 dietary treatments, with six replicate pens of 10 birds per treatment, comprising a basal control diet and 10 selenium-supplemented diets arranged in a 2 × 5 factorial design (Se-Met or L-SeMC at 0.05, 0.10, 0.15, 0.20, or 0.25 mg Se/kg) for 42 days. Selenium supplementation significantly improved average daily gain, final body weight, and feed conversion ratio (*p* < 0.05). Model-estimated stationary points ranged from 0.137 to 0.142 mg/kg for Se-Met and 0.080–0.126 mg/kg for L-SeMC. Using source-specific model-estimated stationary points, L-SeMC showed 1.85- to 1.93-fold higher biological potency than Se-Met. Birds fed L-SeMC exhibited higher plasma glutathione peroxidase activity, lower malondialdehyde, improved meat color (higher a*__24_h), higher ultimate pH, and lower drip loss (*p* < 0.05). L-SeMC is more effective than Se-Met for enhancing broiler growth, antioxidant capacity, and meat quality.

## Simple Summary

Selenium is an essential nutrient for poultry that supports growth, immune function, and meat quality. While selenomethionine is commonly used in chicken feed, a newer form called L-selenomethylselenocysteine may be more effective for selected traits under the conditions tested. We compared these two selenium sources in 660 male broiler chickens over 42 days, testing different amounts of each. Our results show that L-selenomethylselenocysteine was nearly twice as effective as selenomethionine at improving chicken growth, reducing oxidative stress, and enhancing meat quality, including better color and reduced water loss during storage. These findings suggest that L-selenomethylselenocysteine could be a superior choice for poultry nutrition, potentially producing healthier chickens and selenium-enriched meat that benefits human nutrition.

## 1. Introduction

Selenium (Se) is an essential micronutrient for human health, yet dietary selenium deficiency remains a global public health concern affecting over one billion people worldwide. Food animal production represents an effective strategy for selenium biofortification to enhance dietary selenium intake. Broilers, as one of the most consumed meat sources globally, offer rapid growth rates and high feed efficiency, making them an ideal model for evaluating selenium supplementation strategies aimed at producing selenium-enriched meat. Selenium, an essential trace element in poultry nutrition, exerts biological effects that strongly depend on its chemical form and dietary inclusion level [1]. It functions primarily by being incorporated into the active site of selenoproteins—such as glutathione peroxidase (GSH-Px)—which play a central role in the antioxidant defense system [2]. By neutralizing peroxides and maintaining cellular redox homeostasis, Se helps protect tissues from oxidative damage and supports overall physiological function [3].

Dietary selenium supplementation may improve broiler growth performance, including average daily gain (ADG) and feed conversion ratio (FCR), depending on basal selenium status, supplementation level, and selenium source [4,5]. These potential benefits may be associated with reduced oxidative stress, enhanced immune competence, and the regulation of metabolic enzymes involved in nutrient utilization [6]. Selenium deposition in muscle may also enhance endogenous antioxidant capacity and retard postmortem lipid and protein oxidation. These effects may help maintain meat color stability, water-holding capacity, pH, and tenderness, thereby improving meat quality and storage stability [7,8].

Under experimentally induced selenium-deficient conditions, poultry may exhibit growth retardation, poor feather development, degenerative lesions in cardiac and skeletal muscles, impaired immune responses, and reduced reproductive performance [3,9,10,11]. Such deficiency can also decrease GSH-Px activity and increase muscle MDA content, resulting in oxidative damage [12]. These findings illustrate the importance of meeting dietary selenium requirements but should not be interpreted as typical responses under adequately supplemented commercial conditions.

Dietary selenium is primarily supplied in inorganic and organic forms, which differ in their absorption and metabolic utilization. Inorganic selenium, commonly provided as sodium selenite or sodium selenate, remains widely used because of its low cost, acceptable efficacy, and regulatory approval. However, its utilization may be influenced by dietary interactions, and it requires metabolic conversion before incorporation into selenoproteins [5,13]. Its relatively high reactivity may also affect nutrient stability during feed processing and storage [14]. Its safety margin also requires careful consideration because excessive supplementation may lead to selenium accumulation and toxicity, with adverse effects on animal health and performance.

In contrast, Se-Met is absorbed through amino acid transport pathways and is generally considered highly bioavailable [15,16]. It can be incorporated into body proteins, allowing selenium to be retained and subsequently released in tissues, which may enhance selenium deposition and bioavailability [17]. Although organic selenium is generally considered highly bioavailable, its safety depends on the supplementation level, and excessive intake of any selenium source may cause toxicity [18]. Numerous studies confirm that organic selenium sources outperform inorganic forms in improving poultry growth performance, antioxidant capacity, and end-product quality [19]. Se-Met is naturally present in plants and widely used as an organic selenium source because of its high bioavailability [20]. Previous research has primarily evaluated its effects on antioxidant capacity, immune function, and stress resilience in livestock and poultry [21]. However, systematic dose–response comparisons between Se-Met and emerging organic selenium sources, such as L-SeMC, remain limited in broilers.

L-Selenomethylselenocysteine (L-SeMC) is a naturally occurring selenoamino acid structurally analogous to L-cysteine [13]. Unlike Se-Met, L-SeMC can be directly cleaved by β-lyase to generate methylselenol and other active selenium metabolites. This distinct metabolic pathway may contribute to its reported antioxidant and anti-inflammatory activities [18,22,23,24], providing a biological rationale for evaluating L-SeMC as a selenium source in broilers. Previous poultry-related research on L-SeMC has examined inflammation-related responses in a chicken macrophage model, indicating potential anti-inflammatory activity [25]. However, L-SeMC has not been systematically compared with Se-Met across multiple dietary levels. Its optimal supplementation level and relative biological potency based on growth performance, antioxidant status, plasma biochemical indices, and meat quality therefore remain unclear. The present study addresses these gaps through a multi-level, head-to-head comparison of L-SeMC and Se-Met in broilers.

Therefore, this study compared the effects of L-SeMC and Se-Met at different supplementation levels on growth performance, antioxidant capacity, plasma biochemical indices, and meat quality in white-feathered broilers. Quadratic regression was used to estimate the optimal supplementation range and relative biological potency of each source. We hypothesized that L-SeMC would exhibit greater biological efficacy than Se-Met because of its distinct metabolic pathway and greater production of active selenium metabolites.

## 2. Materials and Methods

### 2.1. Experimental Design and Animal Management

A total of 660 healthy one-day-old male Cobb 500 broiler chickens (initial body weight, 47.17 ± 0.03 g) were used. After individual weighing, birds were allocated to 66 pens using a computer-generated randomization procedure, with 10 birds per pen. The pens were then randomly assigned to the 11 dietary treatments, with six replicate pens per treatment. No formal a priori power calculation was performed; the sample size was determined based on previous broiler feeding studies and practical considerations. Treatment pens were distributed throughout the poultry house rather than being placed in contiguous treatment blocks to minimize potential location effects. The 42-day trial was divided into a starter phase (days 1–21) and a grower phase (days 22–42). This two-phase feeding program was adopted in accordance with the nutrient recommendations of the NRC (1994) [26] and the Chinese Feeding Standard for Broilers (NY/T 33-2004) [27], which were used for diet formulation in this study. Although current commercial broiler production may employ additional feeding phases, the two-phase program was consistently applied to all treatments to enable a controlled comparison of the effects of selenium source and supplementation level.

The experiment was conducted in a windowed poultry house without access to an outdoor run. Natural ventilation was supplemented with mechanical ventilation to maintain appropriate environmental conditions. Birds were housed in 66 separate floor pens, with 10 birds per pen. Each pen was equipped with a raised plastic-mesh floor approximately 50–60 cm above the ground. No litter or other bedding material was used, and excreta passed through the mesh and accumulated beneath the raised floor. Excreta were removed daily. Birds were group-housed within each pen and had ad libitum access to feed and water. The house temperature was maintained at 30–32 °C for the first 3 days, then gradually reduced to 28–30 °C from days 4–7, and further decreased by approximately 2 °C per week, stabilizing at about 21 °C from day 21 until the end of the trial. Relative humidity was controlled at 60–70%. Artificial lighting was provided using warm-white LED lamps. The daily photoperiod was 24 h on Day 1, gradually reduced from 23 to 18 h during Days 2–7, maintained at 16 h during Days 8–21 and 18 h during Days 22–35, and gradually increased from 18 to 23 h during Days 36–42. Light intensity was maintained at 10–20 lux during the first 3 days and approximately 5 lux thereafter. All dietary treatments received the same vaccines according to an identical vaccination schedule. Birds were vaccinated against Marek’s disease, coccidiosis, Newcastle disease–infectious bronchitis (ND-IB), and infectious bursal disease (IBD) following standard protocols. Specifically, a tetravalent live coccidiosis vaccine (Foshan Zhengdian Biotechnology Co., Ltd., Foshan, China) was administered by spray on Day 1. Daily health checks and mortality records were maintained. All pens were regularly cleaned and disinfected according to farm standard operating procedures. Experimental diets were produced under standardized conditions to avoid moisture, clumping, or mold. Feed intake was recorded per pen at regular intervals.

### 2.2. Diet Composition and Nutrient Levels

The basal diet was formulated as a corn–soybean meal diet without additional selenium. The composition and analyzed nutrient levels of the basal diets for the starter (1–21 days) and grower (22–42 days) phases are detailed in Table 1. The term “without additional selenium” refers to the absence of intentionally added supplemental selenium and does not imply that the basal ingredients were completely selenium-free. The formulation was in accordance with the China Feed Composition and Nutritional Value Table (31st Edition, 2020) and the nutrient recommendations of the NRC (1994) [26] and the Chinese Feeding Standard for Broilers (NY/T33-2004) [27]. NRC (1994) was additionally used as an internationally recognized reference, particularly for comparison with the established selenium recommendation for broilers, rather than as the sole basis for diet formulation.

After mixing, samples of the basal diets were collected, ground through a 1 mm sieve, and stored at −20 °C for chemical analysis to characterize the analyzed nutrient composition. All analyses were performed in triplicate in our laboratory according to the relevant Chinese national standards, and the method detection limit for selenium was 0.01 mg/kg. Specifically, crude protein was determined using the Kjeldahl method according to GB/T 6432-2018 [28], calcium was determined using atomic absorption spectrometry according to GB/T 13885-2017 [30], total phosphorus was determined using spectrophotometry according to GB/T 6437-2018 [31], and amino acids, including lysine, methionine, and cystine, were determined according to GB/T 18246-2019 [29]. The total selenium concentration of the complete mixed basal diets was analyzed according to GB/T 13883-2008 [32]. Metabolizable energy was calculated based on the China Feed Composition and Nutritional Value Table (31st Edition, 2020) [33], whereas non-phytate phosphorus was calculated from the analyzed total phosphorus content. Individual basal ingredients were not analyzed separately for selenium; therefore, the reported value represents the analytical result for the complete mixed basal diet.

The five supplemental selenium levels (0.05, 0.10, 0.15, 0.20, and 0.25 mg Se/kg) were selected as evenly spaced increments of 0.05 mg/kg across a nutritionally relevant range. This range included levels below, at, and above the NRC (1994) [26] recommended selenium requirement for broilers (0.15 mg/kg) and was also informed by previous reports indicating that organic selenium may exert optimal biological effects within or slightly above the recommended range. Thus, the selected levels were expected to cover the ascending, optimal, and potentially plateauing portions of the biological response while remaining well below the maximum tolerable level of 2 mg/kg. The equal increments were chosen to facilitate systematic comparisons between selenium sources and evaluation of dose–response relationships. Dietary treatments consisted of: (1) a control group (CON) fed the basal diet; (2) Se-Met groups supplemented with 0.05, 0.10, 0.15, 0.20, or 0.25 mg Se/kg from selenomethionine (provided by Guangdong Xinnandu Feed Technology Co., Ltd., Guangzhou, China, with a selenium content of ≥40.2%); and (3) L-SeMC groups supplemented with 0.05, 0.10, 0.15, 0.20, or 0.25 mg Se/kg from L-selenomethylselenocysteine (provided by Guangdong Fangshanneng Animal Health Care Co., Ltd., Guangzhou, China, with a selenium content of ≥43.3%). The Se-Met and L-SeMC products were used as the sole supplemental selenium sources in their respective treatment diets, and the listed supplementation levels refer to selenium supplied by the respective products. The total selenium concentration of the complete mixed basal diet, rather than supplemental selenium alone, was measured by atomic fluorescence spectrometry according to GB/T 5009.93-2017 [34]. The method detection limit for selenium was 0.01 mg/kg, and the selenium concentration in the basal diet was below this limit. Thus, “<0.01 mg Se/kg” represents a below-detection-limit analytical result and should not be interpreted as an exact dietary selenium concentration. It also does not indicate the complete absence of selenium. Individual basal ingredients were not analyzed separately for selenium; therefore, this result represents the analyzed complete mixed basal diet rather than the summed selenium concentrations of the individual ingredients. The CON diet contained no intentionally added supplemental selenium, but the actual total dietary selenium concentration available to the birds could not be determined more precisely than the analytical result of below 0.01 mg/kg. Feed samples from all experimental diets were analyzed in triplicate using the same method, and the analyzed selenium concentrations of the experimental diets are presented in Table S1.

### 2.3. Growth Performance Measurement and Calculation

Throughout the trial, daily feed provision and residues per pen, bird health (including appetite and fecal condition), and mortality were recorded. Initial body weight (BW_1d) was measured for all birds upon arrival at 1 day of age. On the mornings (8:00) of days 22 and 42, after a 12 h fast with water available, all surviving birds in each pen were weighed, and the average body weight for each pen was calculated at 22 d (BW_22d) and 42 d (BW_42d), respectively. Average daily gain (ADG), average daily feed intake (ADFI), and feed conversion ratio (FCR) were calculated for the starter (1–21 d), grower (22–42 d), and overall (1–42 d) phases using the following formulas: ADG (g/d) = (Final average BW − Initial average BW)/Days in the phase; ADFI (g/d) = (Total feed offered − Total feed residue)/(Days in the phase × Average number of birds in the phase); FCR = Total feed consumption/Total weight gain. Mortality was recorded daily for each pen. Phase-specific mortality during days 1–21 and days 22–42 was calculated as the number of spontaneous deaths during the respective phase divided by the number of birds present in that pen at the beginning of the phase. Cumulative mortality during days 1–42 was calculated as the total number of deaths during the trial divided by the initial number of birds in each pen. No birds were culled because of disease, and scheduled sampling was not included in the mortality calculations. Mortality was calculated separately for each pen, and the replicate pen was used as the experimental unit for all growth-performance variables, including BW, ADG, ADFI, FCR, and mortality.

### 2.4. Blood Sampling and Biochemical Analysis

On the morning of Day 43, all birds were fasted for 12 h with free access to water. Two broilers per replicate pen with body weights close to the pen average were selected for blood collection via the wing vein, yielding 12 sampled birds per treatment, with two birds sampled from each of six replicate pens (132 birds in total). These sampled birds were used for laboratory measurements, whereas the replicate pen remained the experimental unit for statistical analysis. Blood collection from all selected birds was completed within a 2 h period in the morning to minimize potential circadian variation among treatments. Blood was drawn into 10 mL vacuum tubes containing EDTA-K_2_ anticoagulant and gently inverted. All samples were maintained at room temperature and centrifuged within 30 min after collection under identical processing conditions. Samples were centrifuged at 3000× *g* for 10 min at 4 °C (Eppendorf 5424 R, Hamburg, Germany). The plasma layer was collected, aliquoted into cryovials, and stored at −20 °C until analysis. Following blood collection, the same birds were used for the subsequent slaughter performance and meat quality assessments described below.

Plasma biochemical parameters were measured using an automatic biochemical analyzer (Hitachi 7080, Tokyo, Japan). The following items were determined using commercial kits (Zhongsheng Beikong Bio-tech Co., Ltd., Beijing, China): triglycerides (TG; Cat. No. C110-1), total cholesterol (T-CHO; Cat. No. C111-1), high-density lipoprotein cholesterol (HDL; Cat. No. C112-1), low-density lipoprotein cholesterol (LDL; Cat. No. C113-1), aspartate aminotransferase (AST; Cat. No. C010-2), alanine aminotransferase (ALT; Cat. No. C009-2), total bilirubin (T-Bil; Cat. No. C019-1), creatinine (CRE; Cat. No. C011-1), and uric acid (UA; Cat. No. C012-1).

Plasma antioxidant indices were assessed using kits from the Nanjing Jiancheng Bioengineering Institute (Nanjing, China), including glutathione peroxidase (GSH-Px; A005), malondialdehyde (MDA; A003-1-1), total superoxide dismutase (T-SOD; A001-1-1), total antioxidant capacity (T-AOC; A015-1-1), total glutathione (T-GSH; A061-1-1), oxidized glutathione (GSSG; A061-1-1), and reduced glutathione (GSH; A006-1-1). All procedures followed the manufacturer’s instructions. Each plasma sample was assayed in triplicate for each antioxidant index, and the mean of the three technical replicates was used as the bird-level measurement. The two bird-level measurements from the same pen were then averaged to obtain one pen-level value for statistical analysis. Absorbance was read on a microplate reader (SpectraMax M2, Molecular Devices, Sunnyvale, CA, USA). Total glutathione (T-GSH) was determined via the DTNB method, and GSSG was measured after GSH elimination. Plasma biochemical and antioxidant indices were measured separately in both sampled birds from each pen. For statistical analysis, the two bird-level measurements from each pen were averaged, and the resulting pen-level mean was used as the observational unit, yielding six independent pen-level observations per treatment.

### 2.5. Slaughter Performance and Meat Quality Assessment

After blood collection, both sampled birds from each replicate pen were euthanized by trained personnel using cervical dislocation. Loss of consciousness was confirmed before the cervical blood vessels were immediately severed to ensure rapid exsanguination. Death was confirmed before tissue collection. The procedure was performed in accordance with the approved institutional animal-use protocol and with reference to the AVMA Guidelines for the Euthanasia of Animals [35] for poultry. All sampled birds were used for the assessment of carcass traits, organ weights, organ indices, and meat quality. Although two birds were measured from each pen, the two bird-level measurements were averaged within the pen before statistical analysis. Thus, 12 birds were sampled per treatment, whereas six pen-level observations were used as the independent experimental units for these measurements. Slaughter body weight (BW_43d) was recorded prior to exsanguination. Immediately after slaughter, the liver, spleen, bilateral pectoralis major, pectoralis minor, and gastrocnemius muscles were excised. Tissues were rinsed with pre-cooled saline, blotted dry, and weighed (Sartorius BSA224S, Göttingen, Germany). Organ indices were calculated as Organ index (%) = [Fresh organ weight (g)/BW_43d (g)] × 100.

Meat quality was evaluated using the left pectoralis major muscle. For drip loss determination, a fresh muscle sample was weighed (W_1_), suspended in a sealed container at 4 °C for 24 h, gently blotted to remove surface moisture, and reweighed (W_2_). Drip loss was calculated as [(W_1_ − W_2_)/W_1_] × 100. For shear force measurement, a separate portion of the same muscle was trimmed into a 6 cm × 3 cm × 3 cm block, vacuum-packed, and heated in a 72 °C water bath until its geometric center reached 70 °C. After cooling to room temperature, the cooked sample was cut into strips parallel to the muscle fiber direction. Shear force was measured perpendicular to the fibers using a texture analyzer (TA.XT Plus, Stable Micro Systems, London, UK) equipped with a Warner–Bratzler blade at a test speed of 1 mm/s, a trigger force of 5 g, and a travel distance of 25 mm. Before testing, the texture analyzer was calibrated for force and height according to the manufacturer’s instructions. The maximum shear force (N) was recorded. For traits involving repeated measurements within the same bird or meat sample, repeated measurements were averaged to obtain one bird-level value. The two bird-level values from each pen were then averaged, and the pen-level mean was used for statistical analysis.

Within 45 min post-slaughter and after 24 h of refrigeration at 4 °C, meat color—expressed as lightness (L*), redness (a*), and yellowness (b*), was measured on the internal cut surface of the left pectoralis major muscle rather than on the bone-side surface using a colorimeter (CR-400, Konica Minolta, Tokyo, Japan; D65 light source, 10° observer). Before each measurement, surface moisture was gently removed. The colorimeter had an 8 mm diameter measurement aperture, corresponding to a measurement area of approximately 0.50 cm^2^. Three nonoverlapping readings were taken per sample at sites free of visible blood vessels and fascia. The three readings were treated as technical replicates and averaged to obtain one value for each bird. The two bird-level values from each pen were then averaged to obtain one pen-level value for statistical analysis. At the same time points, muscle pH was measured using a portable pH meter (PB-10, Sartorius, Göttingen, Germany). The electrode was inserted at least 1 cm into the sample, and the reading was recorded after stabilization. The pH meter was calibrated before each use using pH 4.0 and 7.0 standard buffer solutions (Sigma-Aldrich, St. Louis, MI, USA). All meat-quality measurements were performed by the same trained operator.

### 2.6. Statistical Analysis

Body weight, feed intake, ADG, ADFI, and FCR were calculated on a pen basis; therefore, the replicate pen was considered the experimental unit for growth-performance analysis, with six replicate pens per treatment. For plasma biochemical parameters, antioxidant indices, carcass traits, organ weights, organ indices, and meat-quality traits, the two sampled birds from each pen were measured separately, but their bird-level measurements were averaged within the pen before statistical analysis. Thus, six pen-level observations per treatment were used for statistical inference. For antioxidant indices, the three technical replicates for each sample were averaged before obtaining the bird-level measurement. For meat color, the three readings obtained from each muscle sample were treated as technical replicates and averaged to obtain one value per bird; the two bird-level values from each pen were then averaged to obtain the pen-level value used for analysis. Data were analyzed using the replicate pen as the experimental unit and are expressed as mean ± SEM, with SEM values calculated from the six pen-level observations per treatment. Descriptive statistics and ANOVA based on pen-level observations were performed using GraphPad Prism 9.5.0 (GraphPad Software, San Diego, CA, USA), whereas quadratic regression, bootstrap resampling, and correlation analyses were conducted using R software (version 4.3.2). Normality and homogeneity of variances were assessed using the Shapiro–Wilk test and Brown–Forsythe test, respectively.

To evaluate the overall effect of selenium supplementation, all 11 treatments were analyzed by one-way ANOVA using pen-level observations, followed by Dunnett’s test to compare each selenium-supplemented treatment with the unsupplemented control (CON). To examine the main and interactive effects of Se source and supplementation level, only the 10 selenium-supplemented treatments were included in a two-way ANOVA arranged as a 2 × 5 factorial design, with Se source (Type), supplementation level (Level), and their interaction (Type × Level) included in the model. CON was excluded from the factorial analysis because it received no supplemental Se and therefore had no corresponding Se-source classification within the 2 × 5 factorial design. Nevertheless, CON was retained in the one-way ANOVA and served as the reference group in Dunnett’s comparisons. When the interaction was not significant (*p* > 0.05), the main effects were interpreted. Significant main effects (*p* < 0.05) were further analyzed using Tukey’s test. For significant interactions (*p* < 0.05), simple-effects analysis was conducted to assess level differences within each source and source differences at each level.

The significance test results for quadratic regression trends across all evaluated parameters are provided in Table S2. For selected parameters of biological interest, quadratic regression models were fitted separately for Se-Met and L-SeMC to further evaluate dose–response relationships with Se supplementation level: *Y* = β_0_ + β_1_*X* + β_2_*X*^2^, where *Y* is the response variable and *X* is the Se supplementation level. For each source-specific regression, CON was included as the common 0 mg Se/kg baseline, together with the five supplementation levels of the corresponding Se source. Thus, CON was excluded only from the 2 × 5 factorial analysis and was retained in the dose–response regression analysis. The same CON observations were used as the common biological baseline for the two separate source-specific regressions and were not duplicated within either model. Quadratic regression was selected as an exploratory approach because it allowed potential curvilinear responses and an intermediate maximum or minimum within the tested supplementation range to be characterized. More complex nonlinear models were not considered appropriate because only six dose levels, including the control, were available for each selenium source and no specific asymptotic response pattern was assumed. Model performance was evaluated using the overall regression significance, the coefficient of determination (R^2^), and graphical residual diagnostics, including residuals versus fitted values, normal Q–Q, scale–location, and residuals versus leverage plots. Residual normality was also evaluated using the Shapiro–Wilk test. Formal lack-of-fit testing and comparison with alternative regression models were not performed; therefore, the derived optimal concentrations were interpreted as exploratory model-based estimates within the tested supplementation range. The optimal concentration (*X*_opt_) was calculated as *X*_opt_ = –β_1_/(2β_2_). X_opt_ was interpreted as a model-estimated optimum only when the direction of curvature was biologically consistent with optimization and the estimated value was within the tested supplementation range. A bootstrap resampling approach (B = 5000) was applied to estimate the 95% confidence interval of *X*_opt_ and the model’s coefficient of determination (R^2^).

Associations among parameters were assessed using Spearman’s rank correlation analysis based on paired pen-level observations across all 11 dietary treatments. Growth-performance variables were obtained directly at the pen level. For variables measured in two birds per pen, technical replicates were first averaged within each sample where applicable, and the two bird-level measurements were subsequently averaged to obtain one value per pen. Accordingly, each replicate pen contributed one paired observation for each variable, resulting in 66 paired pen-level observations for each correlation (11 treatments × 6 replicate pens per treatment). The analysis was implemented in R using the stats package, and Spearman’s rank correlation coefficient (r_s_) and the corresponding *p*-value were reported. These correlations were interpreted as associations among pen-level responses across the experimental treatment range and not as evidence of causal relationships. The significance level for all tests was set at α = 0.05. The relative biological potency of L-SeMC compared with Se-Met was estimated using the nonlinear multiple-source model described by Littell et al. [36]: *y* = *a* + *b*_0_ [1 − *exp*(*b*_1_*X*_1_ + *b*_2_*X*_2_)], where y is the pen-level response, a is the response at zero supplemental Se, b_0_ is the common response amplitude, and X_1_ and X_2_ are the supplemental Se concentrations provided by Se-Met and L-SeMC, respectively. The coefficients b_1_ and b_2_ represent the response rates for the two Se sources, and the ratio b_2_/b_1_ estimates the biological potency of L-SeMC relative to Se-Met, which was assigned a value of 1.00. This model assumes that the two Se sources share a common response at zero supplementation and follow a common monotonic asymptotic response function, while differing in their dose–response rates. Under these assumptions, the relative potency is treated as constant over the tested supplementation range. The model was selected because it allows the two Se sources to be evaluated simultaneously using a common baseline and response function and provides a direct relative-potency estimate from the ratio of the source-specific response-rate coefficients. By comparison, commonly used Emax, logistic, or Gompertz models are primarily intended to describe dose–response curves and would generally require additional source-specific parameters, such as separate asymptotes, slopes, or inflection points, to compare the two sources. Given the five positive supplementation levels for each source and the common unsupplemented control, fitting a more highly parameterized model was not considered sufficiently supported by the experimental design. Model fit was summarized using the fitted equations and coefficient of determination (R^2^). The model was fitted to 66 pen-level observations comprising six replicate pens within each of the 11 dietary treatments. The relative-potency estimates were calculated as the ratio of the source-specific response-rate coefficients (b_2_/b_1_), with Se-Met assigned a reference value of 1.00. The 95% confidence intervals for the relative-potency estimates were obtained by nonparametric bootstrap resampling at the pen level using 5000 resamples, with the nonlinear model refitted for each bootstrap sample; the confidence limits were defined by the 2.5th and 97.5th percentiles of the bootstrap distribution. Because the common-baseline, common-response-function, and constant-relative-potency assumptions could not be established definitively over the limited supplementation range, the estimated potency values were interpreted as model-dependent estimates applicable to the experimental conditions and dose range examined. The model was implemented in R (version 4.3.2).

## 3. Results

### 3.1. Source- and Level-Dependent Growth-Performance Responses and Model-Estimated Stationary Points

No statistically significant differences were detected in initial body weight among treatments at day 1 (*p* > 0.05). Growth performance data during the starter (1–21 d), grower (22–42 d), and overall (1–42 d) phases are presented in Table 2. Compared with CON, dietary selenium supplementation resulted in statistically significant improvements in selected growth-performance variables, particularly during the starter phase and across the overall 42 d period (Table 2). During the starter phase (1–21 d), 0.15 mg Se/kg Se-Met significantly increased BW_21d and ADG, while 0.15 mg Se/kg Se-Met and 0.15 and 0.20 mg Se/kg L-SeMC resulted in significantly lower FCR values than CON (*p* < 0.05; Table 3).

Over the entire 42 d trial, all supplemented groups except the two 0.25 mg Se/kg groups showed significantly higher BW_42d and ADG than CON (*p* < 0.05). For the two 0.25 mg Se/kg groups, no statistically significant differences from CON were detected for BW_42d or ADG under the conditions of the experiment. Overall FCR (1–42 d) was significantly lower than that of CON in all selenium-supplemented groups except the 0.20 and 0.25 mg Se/kg Se-Met groups. For these two Se-Met groups, no statistically significant difference from CON was detected for overall FCR. In addition, 0.05 mg Se/kg L-SeMC reduced total mortality (1–42 d) relative to CON (*p* < 0.05).

To further distinguish the effects of selenium source and supplementation level, the CON group was excluded and the remaining 10 supplemented groups were analyzed by two-way ANOVA. Supplementation level affected BW_22d, BW_42d, ADG and FCR during 1–21 d, and ADG during 1–42 d (*p* < 0.05), whereas no statistically significant main effects of selenium source or source × level interactions were detected for the remaining growth-performance variables (*p* > 0.05). These nonsignificant results indicate that a statistically detectable source effect or source × level interaction was not demonstrated under the conditions of the experiment; they do not establish equivalence between selenium sources.

Quadratic regression analysis was then used to obtain model-derived estimates of the supplementation levels associated with the stationary point for each selenium source. Only variables showing significant quadratic responses are presented in Table 4, whereas the results for all tested variables are provided in Table S2. For each response variable, the stationary point was calculated from the fitted quadratic regression equation, and the corresponding 95% confidence interval is reported in Table 4. Across the response variables listed in Table 4, the model-estimated stationary points ranged from 0.137 to 0.142 mg Se/kg for Se-Met and from 0.080 to 0.126 mg Se/kg for L-SeMC. These ranges summarize the minimum and maximum point estimates across the significant response variables and should not be interpreted as confidence intervals or as unified optimal supplementation ranges. The stationary-point estimates for L-SeMC were numerically lower than those for Se-Met across the response variables shown in Table 4. However, because several quadratic models had relatively low R^2^ values and the precision of the estimates varied among response variables, these model-estimated stationary points and their confidence intervals should be regarded as preliminary, outcome-specific estimates within the tested supplementation range, rather than as definitive nutritional recommendations.

The levels of 0.10 mg Se/kg for L-SeMC and 0.15 mg Se/kg for Se-Met were selected for the subsequent focused comparisons because they were the tested concentrations closest to the respective model-derived estimates. Accordingly, subsequent analyses focused on the CON group and these two supplementation groups. This selection was used to facilitate comparisons at experimentally tested levels and should not be interpreted as establishing these concentrations as definitive recommendations for commercial broiler production.

### 3.2. Selenium Source and Level Interactively Affect Slaughter Performance

Dietary selenium supplementation resulted in statistically significant differences in selected slaughter traits in broilers (Table 5). Compared with the CON group, BW_43d was higher in all supplemented groups except the 0.25 mg Se/kg L-SeMC group (*p* < 0.05). Liver index was reduced in the 0.25 mg Se/kg Se-Met group and in the 0.10 and 0.20 mg Se/kg L-SeMC groups, whereas spleen index was decreased only in the 0.25 mg Se/kg Se-Met group. No statistically significant differences were detected in pectoralis muscle or gastrocnemius muscle indices between the supplemented groups and CON; however, these results do not establish that the supplementation treatments had no biological effects on these traits.

After excluding the CON group, two-way ANOVA showed that supplementation level and the source × level interaction significantly affected BW_43d (*p* < 0.05). Spleen index was influenced only by supplementation level, whereas pectoralis muscle index showed a significant source × level interaction (*p* < 0.05). No statistically significant effects were detected for liver or gastrocnemius muscle indices.

**Figure 1:**
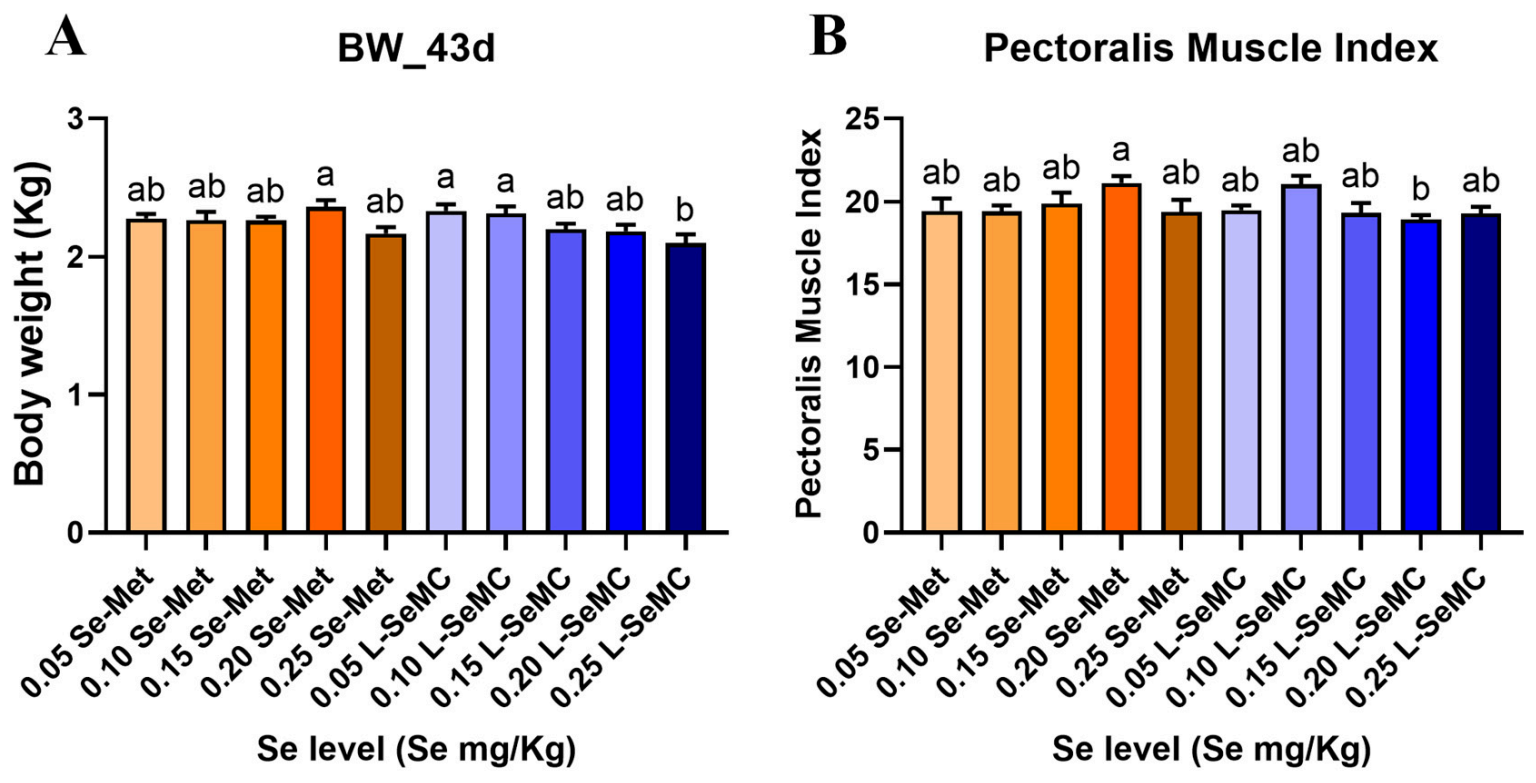
Effects of selenium source and supplementation level on body weight at 43 days and pectoralis muscle index in broilers. (**A**) Body weight at 43 days of age (BW_43d). (**B**) Pectoralis muscle index. Data are presented as mean ± SEM. For variables with a significant selenium source × supplementation level interaction, simple-effects analyses were performed to compare supplementation levels within each selenium source and selenium sources at the same supplementation level. Different lowercase letters indicate significant differences within these specified comparisons (*p* < 0.05).

Because the selenium source × supplementation level interactions were significant for BW_43d and pectoralis muscle index, simple-effects analyses were performed (Figure 1A,B). For BW_43d (Figure 1A), 0.20 mg Se/kg Se-Met was higher than 0.25 mg Se/kg Se-Met, and 0.05 and 0.10 mg Se/kg L-SeMC were higher than 0.25 mg Se/kg L-SeMC. At 0.20 mg Se/kg, Se-Met produced a higher BW_43d than L-SeMC. For pectoralis muscle index (Figure 1B), 0.10 mg Se/kg L-SeMC was higher than 0.15, 0.20, and 0.25 mg Se/kg L-SeMC. At 0.10 mg Se/kg, L-SeMC resulted in a higher pectoralis muscle index than Se-Met, whereas the opposite was observed at 0.20 mg Se/kg.

### 3.3. Selenium Source Influences Key Plasma Enzymes and Interacts with the Level of Creatinine

Plasma biochemical analysis showed statistically significant differences in selected indicators of liver and kidney function among some dietary treatments (Table 6). Compared with the CON group, AST activity was lower in the 0.15 mg Se/kg Se-Met group and in the 0.10–0.25 mg Se/kg L-SeMC groups (*p* < 0.05), whereas ALT activity increased only in the 0.25 mg Se/kg L-SeMC group. UA concentration was reduced in the 0.25 mg Se/kg Se-Met group and in the 0.05, 0.10, and 0.25 mg Se/kg L-SeMC groups (*p* < 0.05). No statistically significant differences were detected for TG, T-CHO, HDL, LDL, or T-Bil under the conditions of the experiment.

After exclusion of the CON group, two-way ANOVA showed significant main effects of selenium source on AST and ALT (*p* < 0.05), with no significant effects of supplementation level or source × level interaction. For CRE, both selenium source and the source × level interaction were significant (*p* < 0.05), whereas no statistically significant effects were detected for the remaining plasma biochemical variables.

**Figure 2:**
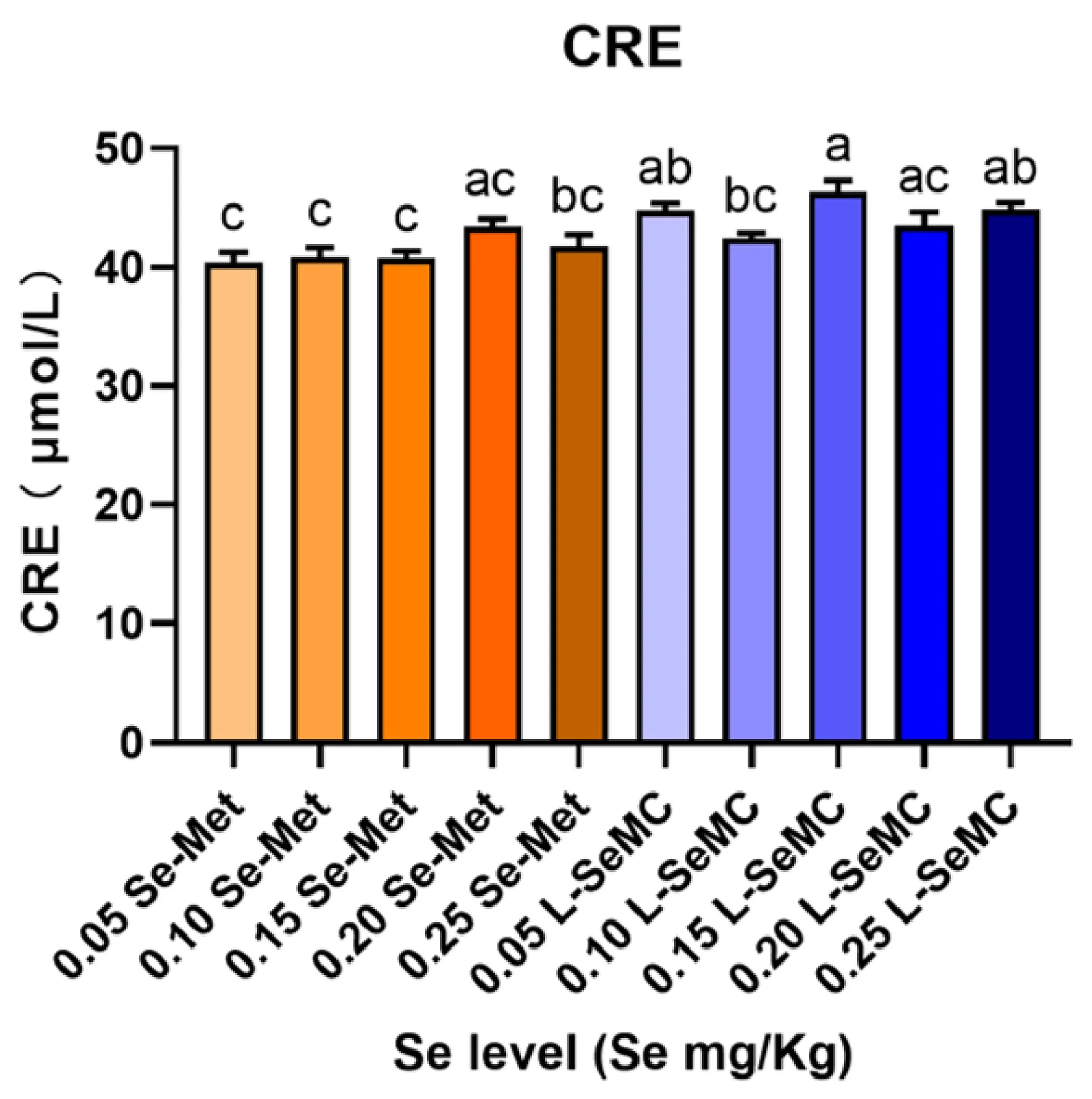
Effects of selenium source and supplementation level on plasma creatinine (CRE) concentration in broilers. Data are presented as mean ± SEM. Because a significant selenium source × supplementation level interaction was detected, simple-effects analyses were performed to compare supplementation levels within each selenium source and selenium sources at the same supplementation level. Different lowercase letters indicate significant differences within these specified comparisons (*p* < 0.05).

Because CRE showed a significant selenium source × supplementation level interaction, simple-effects analysis was performed to compare levels within each source and sources at the same level (Figure 2). Within the L-SeMC group, 0.10 mg Se/kg resulted in significantly lower CRE than 0.15 mg Se/kg. At 0.05, 0.15, and 0.25 mg Se/kg, CRE was higher in the L-SeMC group than in the corresponding Se-Met group.

### 3.4. L-SeMC Demonstrates Superior Antioxidant Efficacy Compared to Se-Met

Dietary selenium supplementation resulted in statistically significant changes in selected plasma antioxidant variables in broilers (Table 7). Compared with the CON group, GSH-Px activity was increased in all selenium-supplemented groups (*p* < 0.05). MDA content was reduced in the 0.15 and 0.25 mg Se/kg Se-Met groups and in the 0.05, 0.10, and 0.20 mg Se/kg L-SeMC groups (*p* < 0.05). T-AOC was higher in the 0.10 and 0.15 mg Se/kg L-SeMC groups than in the CON group (*p* < 0.05).

After exclusion of the CON group, two-way ANOVA showed significant main effects of selenium source, supplementation level, and their interaction on GSH-Px (*p* < 0.05). For MDA, the main effect of selenium source and the source × supplementation level interaction were significant (*p* < 0.05), whereas the main effect of supplementation level was not significant (*p* = 0.56). For T-SOD, T-AOC, and GSSG, only the main effect of selenium source was significant (*p* < 0.05), whereas GSH was affected by both selenium source and supplementation level (*p* < 0.05). No statistically significant effects were detected for the remaining antioxidant indices under the conditions of the experiment.

**Figure 3:**
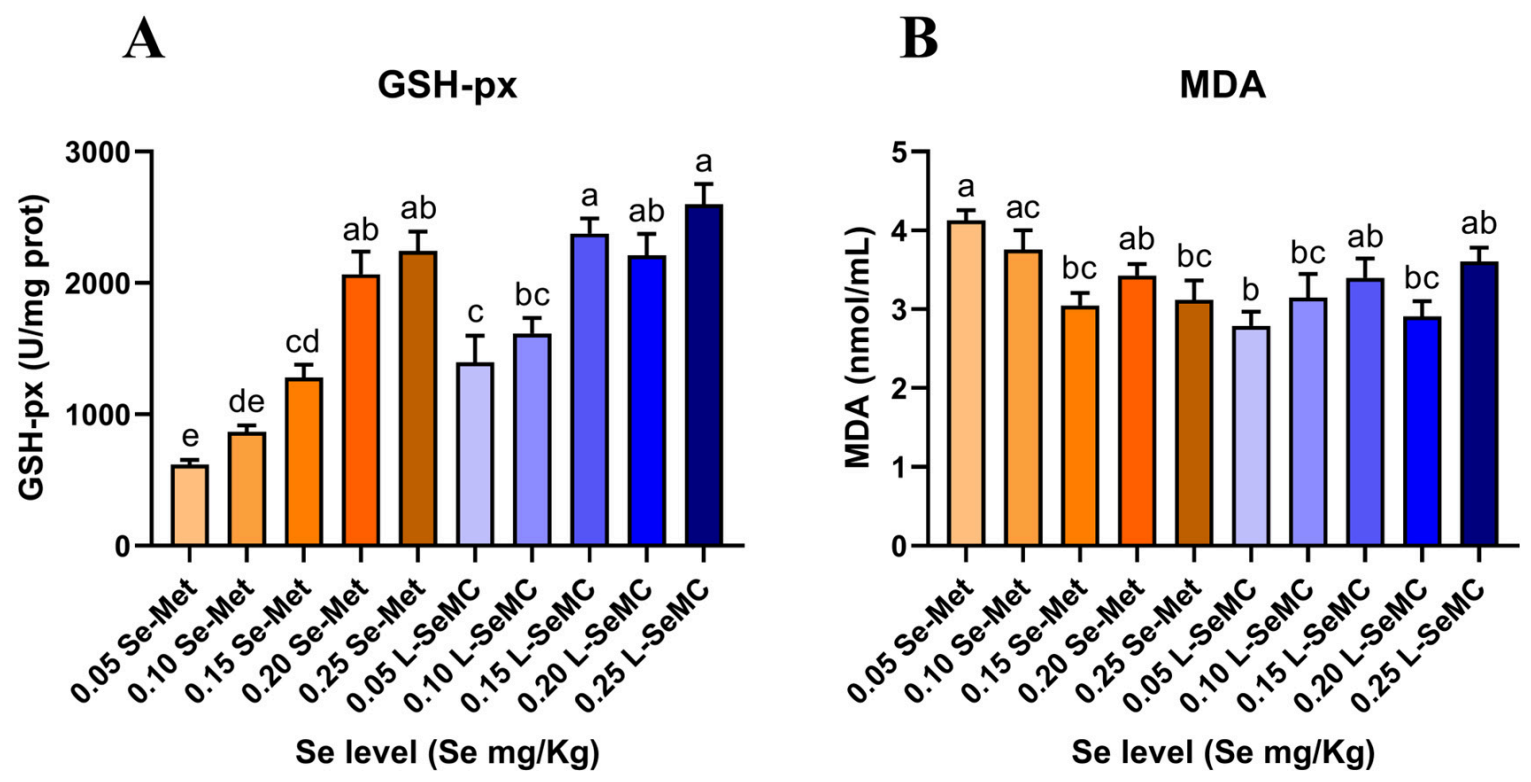
Effects of selenium source and supplementation level on plasma antioxidant indices in broilers. (**A**) Plasma glutathione peroxidase (GSH-Px) activity. (**B**) Plasma malondialdehyde (MDA) concentration. Data are presented as mean ± SEM. For variables with a significant selenium source × supplementation level interaction, simple-effects analyses were performed to compare supplementation levels within each selenium source and selenium sources at the same supplementation level. Different lowercase letters indicate significant differences within these specified comparisons (*p* < 0.05).

Because GSH-Px and MDA showed significant selenium source × supplementation level interactions, simple-effects analyses were performed (Figure 3). GSH-Px activity (Figure 3A) increased with supplementation level for both selenium sources, and L-SeMC produced higher values than Se-Met at 0.05–0.15 mg Se/kg. For MDA (Figure 3B), within the Se-Met treatments, the values at 0.15 and 0.25 mg Se/kg were lower than that at 0.05 mg Se/kg. Between selenium sources, L-SeMC resulted in lower MDA values than Se-Met at both 0.05 and 0.25 mg Se/kg.

### 3.5. Dietary Selenium Improves Meat Color and pH Stability via Source- and Level-Dependent Interactions

Dietary selenium supplementation resulted in statistically significant differences in selected breast-meat color and pH variables after 24 h of refrigerated storage (Table 8). Compared with the CON group, a*_24h and pH_24h were higher in most supplemented groups (*p* < 0.05). L*_24h differed significantly from CON in all groups except the 0.10 mg Se/kg Se-Met group, for which no statistically significant difference from CON was detected. Initial L* was reduced only in the 0.25 mg Se/kg Se-Met and 0.10 mg Se/kg L-SeMC groups.

After exclusion of the CON group, two-way ANOVA showed significant source × level interactions for all 24 h meat quality traits (L*_24h, a*_24h, b*_24h, and pH_24h; *p* < 0.05). A significant main effect of selenium source was detected for L*_24h, whereas supplementation level significantly affected b*_24h and pH_24h. No statistically significant effects were detected for the remaining meat-quality parameters.

**Figure 4:**
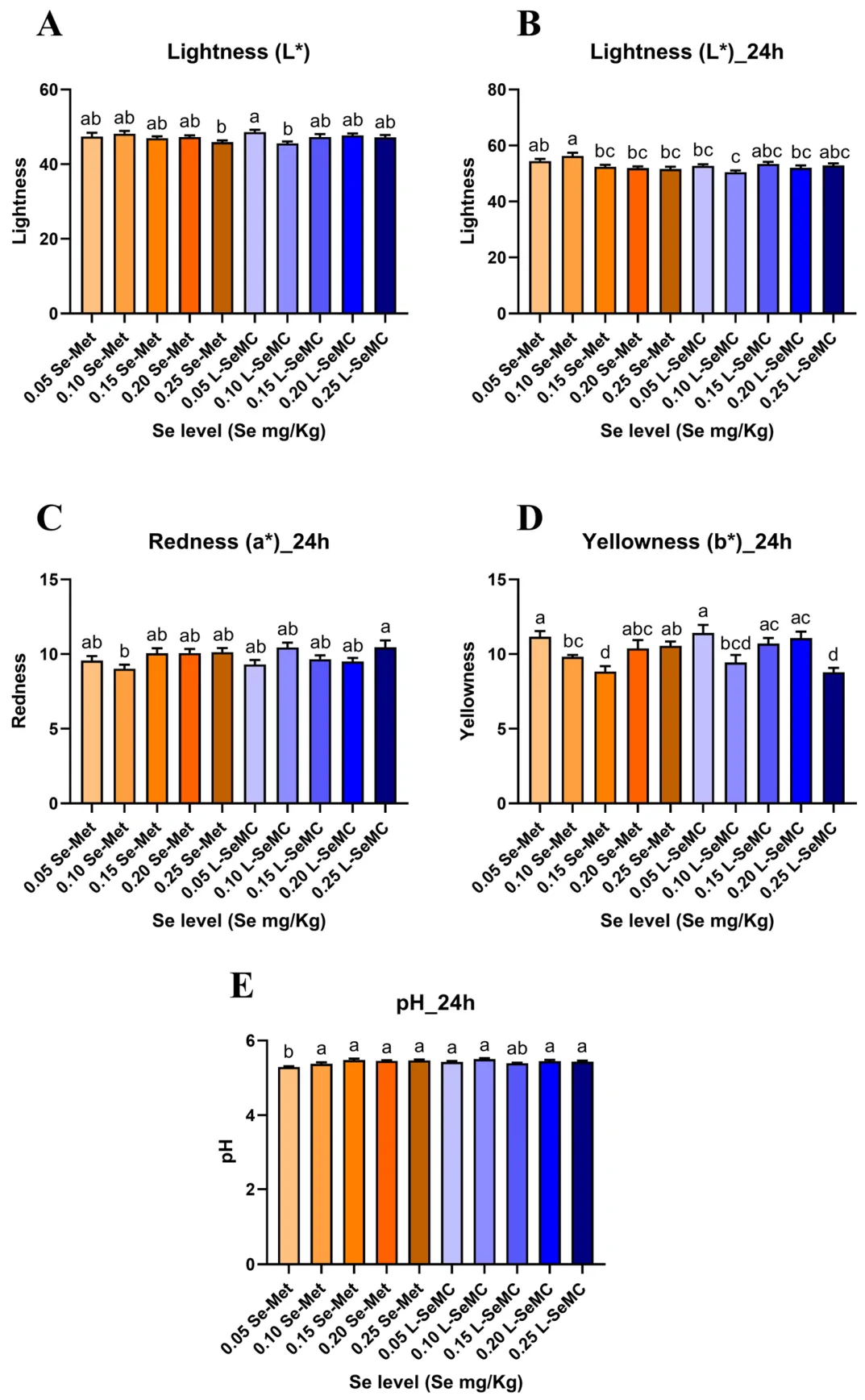
Effects of selenium source and supplementation level on meat color and pH-related traits in broilers. (**A**) Lightness (L*) at 0 h postmortem. (**B**) Lightness (L*_24h) at 24 h postmortem. (**C**) Redness (a*_24h) at 24 h postmortem. (**D**) Yellowness (b*_24h) at 24 h postmortem. (**E**) pH at 24 h postmortem (pH_24h). Data are presented as mean ± SEM. Because the selenium source × supplementation level interaction was significant, simple-effects analysis was performed among the 10 selenium-supplemented treatment combinations. Different lowercase letters indicate significant differences among treatment means based on simple-effects analysis (*p* < 0.05).

Because several post-storage traits showed significant interactions, simple-effects analysis was performed (Figure 4). For L* (Figure 4A) and L*_24h (Figure 4B), 0.10 mg Se/kg L-SeMC produced lower values than the corresponding Se-Met treatment, and within the L-SeMC group, 0.10 mg Se/kg was lower than selected adjacent levels. For a*_24h (Figure 4C), 0.10 mg Se/kg Se-Met was lower than 0.15 and 0.25 mg Se/kg Se-Met, whereas 0.10 and 0.25 mg Se/kg L-SeMC were higher than 0.05 mg Se/kg L-SeMC; at 0.10 mg Se/kg, L-SeMC showed higher a*_24h than Se-Met. For b*_24h (Figure 4D), 0.15 mg Se/kg Se-Met was lower than 0.05, 0.20, and 0.25 mg Se/kg Se-Met, whereas 0.10 and 0.25 mg Se/kg L-SeMC were lower than the other L-SeMC levels; L-SeMC was higher than Se-Met at 0.15 mg Se/kg but lower at 0.25 mg Se/kg. For pH_24h (Figure 4E), 0.15–0.25 mg Se/kg Se-Met resulted in higher values than 0.05 mg Se/kg Se-Met, and L-SeMC showed higher pH_24h than Se-Met at 0.05 and 0.10 mg Se/kg.

### 3.6. Model-Estimated Relative Potency of L-SeMC Compared with Se-Met

The model-estimated relative potency ratio of L-SeMC compared with Se-Met was estimated using BW_43d and plasma GSH-Px activity (Table 9). The nonlinear model was fitted using 66 pen-level observations, comprising six replicate pens within each of the 11 dietary treatments. Based on BW_43d, the estimated relative potency was 1.85 (95% CI: 1.65–2.35), whereas the estimate based on plasma GSH-Px activity was 1.93 (95% CI: 1.52–2.21). The fitted equations and R^2^ values are provided in Table 9, and the 95% confidence intervals were calculated using pen-level bootstrap resampling. These results indicate higher model-estimated response rates for L-SeMC than for Se-Met for the two measured outcomes. However, the estimates are outcome-specific and model-dependent and should not be interpreted as evidence of general biological superiority of L-SeMC.

### 3.7. Exploratory Correlations Between Antioxidant Indices, Growth Performance, and Meat Quality

**Figure 5:**
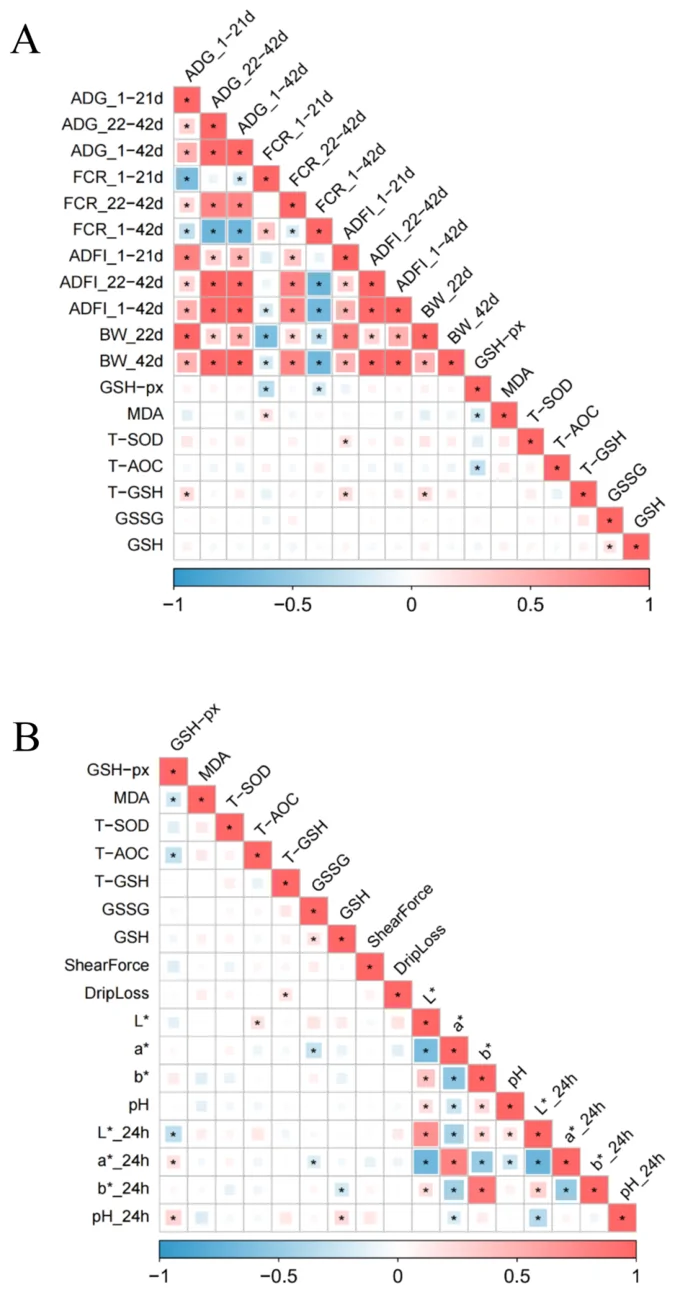
Correlations of plasma antioxidant indices with growth-performance and meat-quality traits in broilers. (**A**) Spearman correlation heatmap between plasma antioxidant indices and growth performance-related variables. (**B**) Spearman correlation heatmap between plasma antioxidant indices and meat-quality traits. Correlations were calculated using 66 paired pen-level observations across the 11 dietary treatments (six replicate pens per treatment). For variables measured in two birds per pen, the bird-level values were averaged within the pen before analysis. Each pen contributed one observation per variable. The color scale represents Spearman’s rank correlation coefficient (r_s_). Asterisks indicate statistical significance (*p* < 0.05). Correlations indicate associations and should not be interpreted as evidence of causality.

Exploratory Spearman correlation analysis was performed to assess the covariation of plasma antioxidant indices with growth performance and meat quality across the experimental treatment structure (Figure 5). Plasma antioxidant indices showed several statistically significant correlations with growth performance (Figure 5A). T-GSH was positively correlated with ADG (1–21 d), ADFI (1–21 d), and BW_22d (*p* < 0.05). GSH-Px activity was negatively correlated with FCR during both 1–21 d and 1–42 d, whereas MDA was positively correlated with FCR during 1–21 d (*p* < 0.05). T-SOD activity was positively correlated with ADFI during 1–21 d (*p* < 0.05).

Plasma antioxidant indices also showed several statistically significant correlations with meat quality traits (Figure 5B). GSH-Px activity was positively correlated with a*_24h and pH_24h, but negatively correlated with L*_24h (*p* < 0.05). T-AOC was positively correlated with L* at slaughter. GSSG was negatively correlated with a* and a*_24h, whereas GSH was negatively correlated with b*_24h and positively correlated with pH_24h (*p* < 0.05). These correlations were exploratory and do not establish an intrinsic biological association or causal relationship between the variables.

## 4. Discussion

### 4.1. L-SeMC Enhances Growth Performance More Efficiently than Se-Met at Lower Supplementation Levels

Selenium is an essential trace element whose biological efficacy varies with its chemical form, a key focus in current nutritional research. This study compared the effects of Se-Met and L-SeMC on broiler performance, providing experimental evidence for the scientific selection of selenium sources. Under the conditions of the present study, supplementation with Se-Met or L-SeMC affected selected growth-performance variables, although the magnitude and direction of the responses varied with Se source and supplementation level. These findings should not be interpreted as evidence that Se supplementation universally improves broiler growth, because responses may depend on basal dietary Se status, diet composition, environmental stress, bird health status, Se source, and supplementation level. Previous studies have also reported improvements in final body weight or feed conversion efficiency following Se supplementation [37,38,39,40]. In the present study, L-SeMC showed more favorable responses than Se-Met for selected ADG and FCR comparisons at certain supplementation levels, including some lower supplementation levels. These findings are consistent with the reported effects of selenium yeast on broiler BW and FCR [4] and suggest that selenium source may contribute to variation in growth responses under specific experimental conditions. However, the biological processes responsible for these source-related differences were not directly evaluated in the present study.

The total selenium concentration of the complete mixed basal diet was below the analytical detection limit of 0.01 mg Se/kg before supplementation. Thus, the reported value of <0.01 mg Se/kg represents a below-detection-limit analytical result rather than an exact dietary selenium concentration. Individual basal ingredients were not analyzed separately for selenium; therefore, the contribution of each ingredient to the background selenium concentration could not be determined, and the reported value refers to the analyzed complete mixed basal diet. The CON diet contained no intentionally added supplemental selenium, but the analytical result does not provide a direct estimate of the amount of selenium biologically available to the birds. Moreover, the initial selenium status of the chicks was not assessed by measuring plasma, liver, muscle, or other tissue selenium concentrations. Therefore, the present study cannot establish that the birds were physiologically selenium-deficient, because physiological deficiency requires biological evidence beyond a low analytical selenium concentration in the diet. The possibility that the low, unquantified background selenium concentration enhanced the apparent response to supplementation remains a plausible hypothesis, but this explanation was not directly tested. Accordingly, responses relative to CON should be extrapolated cautiously to diets with different background selenium concentrations or to commercial production conditions. Comparisons between Se-Met and L-SeMC at matched supplementation levels are more directly interpretable within this experiment because both sources were added to the same basal diet, although differences in their biological handling were not measured.

The more favorable responses to L-SeMC observed in selected comparisons may be biologically plausible in light of reported differences in the handling of organic selenium forms, but the underlying processes were not tested in the present study. Previous studies have suggested that a portion of Se-Met may be incorporated nonspecifically into body proteins [13], whereas L-SeMC has been proposed to undergo metabolism to monomethylated selenium compounds [22]. These findings provide possible biological context for the observed source-related responses, but they do not demonstrate that Se-Met incorporation, L-SeMC metabolism, or methylselenol formation caused the growth-performance differences observed here. The present study did not measure selenium metabolites, tissue selenium deposition, selenoprotein expression, or the incorporation of Se-Met into body proteins. Therefore, no sequential mechanistic pathway from selenium source to selenium metabolism and then to growth performance can be established from the present data. These mechanisms should be regarded as biologically plausible hypotheses requiring direct verification in future studies.

The lower optima estimated from the quadratic regression models for L-SeMC than for Se-Met should be interpreted cautiously and not as direct evidence of greater metabolic efficiency. These values were model-derived stationary points rather than directly observed experimental optima. Moreover, because the quadratic models had low R^2^ values, these estimates should be considered preliminary. They represent model-dependent stationary points within the tested supplementation range rather than definitive nutritional recommendations for commercial broiler production. Quadratic models were retained because the significant quadratic terms indicated curvilinear responses and allowed estimation of stationary points within the tested range.

In terms of carcass traits, statistically significant effects of selenium source or supplementation level were detected for only a limited number of indices in this study. Although Naylor and Choct [41] previously reported that organic selenium could improve eviscerated yield and breast muscle yield in broilers, in the present trial, only the breast muscle index was significantly higher in the L-SeMC group at 0.10 mg Se/kg, whereas no statistically significant differences were detected consistently for the other measured carcass traits. The observed growth-performance responses may reflect greater overall body growth rather than a consistent redistribution of weight toward specific carcass components; however, this interpretation remains tentative because tissue growth patterns and body-composition variables were not comprehensively assessed. The absence of consistent differences in the other carcass traits could reflect the magnitude of the treatment responses, biological variability among birds, the sensitivity of the selected carcass indices, or limited statistical power to detect small or moderate effects. Carcass yield and relative organ indices may also respond differently from ADG and FCR, and the single end-point measurement may not have captured changes occurring earlier or later during the experimental period. Thus, under the present conditions, the observed growth-performance differences were not accompanied by consistent statistically significant changes in the measured carcass traits, although the nonsignificant findings should not be interpreted as evidence that selenium source or supplementation level had no biological effect on these traits.

The BW and overall FCR observed in this study did not reach the performance objectives for modern Cobb broilers, which may partly reflect differences between the optimized commercial conditions underlying those objectives and the conditions of the present research facility. Other contributors could include variation in environmental conditions, management, health status, and biological variability among birds. The cumulative mortality observed in some groups was higher than that typically reported under commercial conditions. Because systematic postmortem examinations were not performed, the underlying cause could not be determined. Although no statistically significant difference in mortality was detected among treatments, the study was not specifically powered to evaluate mortality; therefore, the absence of significance should not be interpreted as evidence of equivalent mortality risk among treatments. No consistent numerical pattern related to selenium source or supplementation level was observed. This lack of a consistent pattern may reflect biological variability, the relatively small number of replicate pens, differences in treatment magnitude, or other uncontrolled causes of mortality, although these possibilities were not directly tested. Nevertheless, all treatments received the same basal diet and were managed under identical conditions, supporting the internal comparability of the Se-Met and L-SeMC treatments within this experiment. Therefore, these findings should be interpreted as comparative responses under the present experimental conditions, and their applicability to high-performing commercial broilers requires further validation. Another limitation is the absence of an inorganic selenium-supplemented positive control, such as sodium selenite. Therefore, the present results permit direct comparison only between Se-Met and L-SeMC and do not establish their efficacy relative to inorganic selenium sources. Future studies should include an inorganic selenium treatment at matched supplementation levels.

### 4.2. L-SeMC Enhances Antioxidant Defense More Efficiently than Se-Met

Most plasma biochemical variables showed no consistent responses to selenium source or supplementation level, although selected changes were observed in AST, ALT, UA, and CRE. Previous studies have reported changes in lipid-related biochemical indices following selenium supplementation [42], but no statistically significant treatment effects on TG or T-CHO were detected in the present study. The absence of consistent responses in these variables could reflect the combined influence of baseline physiological status, biological variability, treatment magnitude, statistical power, and sampling timing. Plasma biochemical variables are regulated by multiple physiological and nutritional factors, and their responses may remain relatively stable when the treatment effect is modest or when the birds are not experiencing a pronounced metabolic or pathological challenge. Because the birds were not subjected to a specific oxidative, infectious, or heat-stress challenge, the experimental conditions may have limited the magnitude of treatment-related changes in these systemic biochemical indicators. Moreover, the single terminal sampling time may not have captured transient responses. Given the possible limitation in statistical power, these results do not establish the absence of an effect on lipid metabolism. In particular, CRE showed a significant selenium source × supplementation level interaction, consistent with previous reports that selenium supplementation may alter blood CRE concentrations [43,44]. However, plasma CRE can be influenced by renal function, muscle mass, muscle metabolism, and hydration status. Because these factors were not separately assessed, the physiological basis and biological significance of the observed CRE response cannot be determined from the present data. Therefore, these biochemical results are considered complementary physiological information, and the following discussion focuses primarily on antioxidant indices that are commonly used to characterize selenium-associated antioxidant responses.

The principal antioxidant responses observed in the present study were increased plasma GSH-Px activity and reduced MDA concentration. Malondialdehyde is a widely used marker of lipid peroxidation and oxidative damage [45]. Supplementation with Se-Met or L-SeMC significantly reduced plasma MDA content compared with CON, consistent with previous broiler studies that reported reduced lipid peroxidation following selenium supplementation [21,46,47]. In contrast, T-SOD, T-AOC, and glutathione-related indices showed weaker or less consistent treatment responses. The stronger response of GSH-Px may be biologically consistent with its selenium-dependent function; however, the present study did not assess whether changes in GSH-Px activity resulted from altered selenoprotein synthesis or other molecular processes. The other indices represent different components of the antioxidant system and may vary in their sensitivity to selenium source, supplementation level, basal antioxidant status, and sampling time. Therefore, the present results showed selective changes in the measured antioxidant indices rather than a uniform response across all antioxidant variables. The weaker responses of some indices should not be interpreted as evidence that selenium supplementation had no effect on these components, because small treatment effects or transient changes may not have been detected under the present experimental conditions.

Selenium can contribute to antioxidant-related biological processes through selenoproteins such as GSH-Px. In this study, plasma GSH-Px activity was significantly enhanced in the Se-Met- and L-SeMC-supplemented groups, which is consistent with findings reported by Jing et al. [21], Wang and Xu [48], Zhang et al. [49], and Wan et al. [50] in various poultry species. However, the present results show an association between selenium supplementation and plasma GSH-Px activity, but do not establish the molecular basis of this association, because selenium incorporation into selenoproteins and related molecular responses were not measured. Selenium supplementation markedly increased GSH-Px activity, whereas T-SOD activity showed no consistent treatment response. Although GSH-Px and SOD are involved in the handling of different reactive oxygen species and are subject to different regulatory processes, the limited T-SOD response cannot be used to infer that increased GSH-Px activity reduced the demand for SOD. The differential responses instead suggest that the measured antioxidant components may have varied in their sensitivity to selenium supplementation under the present experimental conditions [51,52,53]. The weaker or less consistent responses in T-SOD, T-AOC, GSH, GSSG, and related indices could reflect differences in their regulation and biological specificity, but may also have been influenced by baseline antioxidant status, biological variability, treatment magnitude, sampling time, or limited statistical power. Unlike GSH-Px, T-SOD is not a selenium-dependent enzyme, whereas T-AOC reflects the combined contribution of enzymatic and nonenzymatic antioxidants. GSH and GSSG concentrations are influenced by their synthesis, oxidation, reduction, and utilization and may therefore not change in parallel with GSH-Px activity. These considerations provide possible explanations for the variable-specific responses, but none of them was directly tested in the present study.

As an integrated measure of enzymatic and nonenzymatic antioxidant components, T-AOC provides complementary information on overall antioxidant capacity [54]. Supplementation with the two organic selenium sources increased plasma T-AOC values in the present experiment, aligning with reports by Gong et al. [55] and Zhou et al. [56]. This finding may indicate an improvement in the measured antioxidant capacity, although it does not by itself demonstrate a comprehensive enhancement of antioxidant reserves. These antioxidant responses may be consistent with the involvement of organic selenium in antioxidant-related processes, including changes in GSH-Px activity [57,58], although the relevant molecular pathways were not assessed in the present study. Previous studies have proposed that organic selenium stored in tissues may form a mobile selenium pool that supports antioxidant enzyme synthesis during oxidative stress [13,59]. This proposed tissue selenium pool could provide one possible explanation for changes in antioxidant activity, but tissue selenium deposition, selenium metabolites, and antioxidant enzyme synthesis were not measured in the present study. Therefore, this mechanism remains hypothetical and cannot be inferred from the observed plasma antioxidant responses alone.

Under the present experimental conditions, L-SeMC showed more pronounced responses than Se-Met for selected antioxidant indices, including higher GSH-Px activity at 0.05–0.15 mg Se/kg and lower MDA concentration at 0.05 and 0.25 mg Se/kg. These source-related differences may be biologically consistent with differences in selenium utilization reported for organic selenium compounds [22]. However, selenium metabolites, selenoprotein expression, tissue selenium deposition, and other relevant metabolic variables were not measured. Therefore, the present data do not establish that differences in selenium utilization caused the observed GSH-Px or MDA responses. The observed differences could also reflect variation in treatment magnitude, biological variability, indicator sensitivity, sampling timing, or statistical precision, and the underlying mechanism requires direct investigation.

In summary, supplementation with the two organic selenium sources was associated with changes in selected measured antioxidant indices, with L-SeMC showing more pronounced responses than Se-Met for selected comparisons of GSH-Px activity and MDA concentration under the present experimental conditions. These findings describe source-related differences in the measured indices but do not by themselves establish a general difference in antioxidant efficacy or the mechanism underlying these responses. The magnitude of these responses varied with selenium source, supplementation level, and antioxidant index. Nonsignificant responses in other antioxidant variables should be interpreted as a lack of detected statistical differences under the present conditions rather than as evidence of biological equivalence or complete absence of an effect.

### 4.3. Source- and Level-Dependent Effects of L-SeMC and Se-Met on Meat Quality

This study showed that supplementation with Se-Met or L-SeMC resulted in statistically significant differences in selected meat-quality variables, including meat color stability and pH after 24 h of refrigerated storage, consistent with previous reports [60,61]. The meat-quality responses observed with Se-Met and L-SeMC may be biologically consistent with selenium-associated antioxidant processes. One possible interpretation is that selenium-dependent antioxidant activity, including GSH-Px activity, could influence the handling of peroxides and reactive species during postmortem storage and thereby affect oxidation-related meat-quality measurements [62,63]. However, the present study did not measure selenoprotein synthesis, peroxide removal, reactive-species levels, or lipid and protein oxidation directly; therefore, this proposed pathway remains untested. In our experiment, L-SeMC resulted in lower drip loss and higher a*_24h values at specific supplementation levels than Se-Met groups, aligning with findings by Edens and Sefton [64] and Jiang et al. [12]. Previous studies have reported that organic selenium may be more effective than inorganic selenium in reducing drip loss. One proposed explanation in the literature is that organic selenium, including L-SeMC, may be deposited more efficiently in muscle tissue and could thereby support local antioxidant protection or cell-membrane integrity, potentially reducing fluid exudation [47,65]. However, tissue selenium concentrations, membrane integrity, and local muscle antioxidant activity were not determined; therefore, the present results cannot establish whether selenium deposition or local antioxidant protection contributed to these meat-quality responses.

Meat color stability can be influenced by myoglobin oxidation status. Previous studies have also shown that meat quality and color characteristics may vary with animal- and production-related factors. Dietary factors have also been reported to influence meat-quality traits in livestock species [66], although such findings from ruminants should not be directly extrapolated to broilers. The significantly increased a*_24h values observed in the present study may be consistent with less oxymyoglobin oxidation or lower metmyoglobin formation [67] and may be compatible with greater redness [68,69]. This interpretation is consistent with the findings of Wang et al. [40] in Langshan chickens. However, because metmyoglobin concentration and other direct indicators of myoglobin oxidation were not measured, this interpretation remains a possible explanation rather than a demonstrated mechanism. At the pen level, plasma GSH-Px activity was positively correlated with a*_24h, whereas MDA content was negatively correlated with a*_24h [70]. These correlations should be interpreted as exploratory covariation across the experimental treatment structure rather than as evidence of a general biological association between systemic antioxidant status and muscle oxidative stability [39]. Additionally, L-SeMC at 0.10 mg Se/kg significantly reduced L*_24h, consistent with the findings of Oliveira et al. [71] and Tapiero et al. [72]. However, meat lightness is influenced by multiple factors, including water distribution, surface reflectance, and muscle structure; therefore, the lower L*_24h cannot be attributed solely to myofibrillar contraction or improved water-holding capacity. The observed color differences could also have been influenced by variation in muscle structure, water distribution, treatment magnitude, or measurement timing, although these factors were not separately assessed.

Selenium supplementation also increased pH_24h in the present study. A higher pH_24h may be compatible with a greater distance from the isoelectric point of muscle proteins and could consequently be associated with improved water-holding capacity and reduced drip loss [12,73]. However, protein hydration, protein denaturation, and the distance from the isoelectric point were not directly assessed in the present study. At the pen level, GSH-Px activity was positively correlated with pH_24h, whereas GSSG was negatively correlated with a* and a*_24h [53]. These correlations provide exploratory information on covariation among the measured variables but do not establish a direct relationship between antioxidant status and postmortem meat-quality traits. However, postmortem glycolysis was not measured; therefore, effects on energy metabolism or the rate of pH decline cannot be directly inferred from the present results. Moreover, the correlation analyses were based on 66 paired pen-level observations across the 11 dietary treatments, with each replicate pen contributing one observation per variable. Because these observations comprised six pens nested within each dietary treatment, pooling them across treatments may have captured systematic differences among selenium sources or supplementation levels in addition to within-treatment variation. Although this pen-level analysis provided more information than an analysis based on treatment means, the observed associations may partly reflect common responses of the variables to selenium source and supplementation level. Therefore, the correlations indicate covariation among biological responses across the experimental treatment range but should not be interpreted as evidence of an intrinsic biological association, a predictive relationship, or a direct or causal relationship. Further studies specifically designed to distinguish treatment-driven associations from direct biological relationships are warranted.

In addition, selenium source × supplementation level interactions were detected for selected meat-quality outcomes. For instance, the pattern of shear force responses differed between selenium sources across supplementation levels, which was not fully consistent with reports that organic selenium may improve tenderness [74]. This difference from previous reports could reflect variation in animal breed, management, selenium level, muscle-fiber characteristics, collagen content, or sampling variability [75,76]. Because muscle-fiber and collagen characteristics were not measured, their contributions remain uncertain. The lack of a consistent selenium effect on shear force in the present study warrants further investigation. The variable response pattern could also reflect biological variability, the magnitude of the treatment effects, or limited precision associated with the number of replicate pens.

Across the measured outcomes, statistically significant responses were observed for selected growth-performance, antioxidant, and meat-quality variables, whereas no statistically significant treatment effects were detected for most plasma biochemical parameters, carcass traits, and several antioxidant indices. As discussed above, these variable-specific findings may reflect differences in biological function, physiological regulation, baseline status, response sensitivity, and sampling time. They could also reflect differences in treatment magnitude, biological variability, and statistical precision across outcomes. However, because no a priori power calculation was performed and each treatment included six independent replicate pens, the study may have had limited statistical power to detect small or moderate treatment effects for some outcomes. Consequently, some nonsignificant findings may reflect insufficient precision rather than the absence of biologically relevant differences. Thus, a nonsignificant P-value indicates that a statistically significant difference was not detected under the conditions of the experiment; it does not demonstrate that selenium supplementation had no effect, that the two selenium sources were equivalent, or that any observed numerical differences were biologically unimportant. Accordingly, nonsignificant results should not be interpreted as evidence of biological equivalence or absence of an effect between treatments. Studies with larger numbers of independent replicate pens and repeated sampling are needed to confirm these findings.

Overall, supplementation with Se-Met or L-SeMC resulted in favorable changes in selected measured indices of postmortem oxidative stability and meat quality. Under the present experimental conditions, L-SeMC showed more favorable responses than Se-Met for selected measured indicators, including drip loss, a*_24h, and pH_24h. These findings indicate source-related differences only for the measured outcomes and do not establish broader biological or metabolic superiority of L-SeMC over Se-Met. Although the observed differences may be consistent with source-dependent antioxidant responses, the present study did not directly assess the biological processes that could produce such responses, including tissue selenium deposition, selenium metabolism, selenoprotein expression, and methylselenol production. Therefore, these processes remain possible but untested explanations rather than established mechanisms. The model-estimated relative-potency values should likewise be interpreted as outcome-specific and model-dependent estimates rather than definitive measures of general biological efficacy. These findings are restricted to the two organic selenium sources evaluated and should not be extrapolated to inorganic selenium sources.

The relative-potency estimates should also be interpreted cautiously because they were derived from a restricted nonlinear model fitted to the 66 pen-level observations across the 11 dietary treatments. The model imposed a common baseline and response amplitude for the two selenium sources and assumed that the relative potency remained constant across the tested supplementation range. These assumptions could not be conclusively verified with the present experimental design and dose range. In addition, the model fit was outcome-dependent, with a lower R^2^ for BW_43d (0.622) than for plasma GSH-Px activity (0.947). Therefore, the reported ratios of 1.85 and 1.93 should be regarded as outcome-specific, model-dependent estimates under the present experimental conditions rather than evidence of general biological superiority or a directly measured difference in efficacy between L-SeMC and Se-Met.

## 5. Conclusions

Under the present experimental conditions, L-SeMC showed more favorable responses than Se-Met for selected measured growth-performance, antioxidant, and meat-quality variables at specific supplementation levels in white-feathered broilers. These source-related differences were variable-specific and do not establish general superiority of L-SeMC across all measured outcomes. The quadratic models yielded model-estimated stationary-point ranges of 0.080–0.126 mg Se/kg for L-SeMC and 0.137–0.142 mg Se/kg for Se-Met; however, because several models had relatively low R^2^ values, these ranges should be regarded as preliminary, model-dependent estimates within the tested range rather than definitive nutritional recommendations. Nonsignificant results for other variables should not be interpreted as evidence of no biological effect or equivalence between the selenium sources, particularly because each treatment included only six independent replicate pens. The findings do not establish broader biological, metabolic, or bioavailability superiority of L-SeMC because tissue selenium deposition, selenium metabolism, selenoprotein expression, and methylselenol production were not measured.

## Figures and Tables

**Table 1:** Composition and nutritional levels of the basal diets in this experiment.

Item	1–21 Days	22–42 Days
Corn, %	49.52	56.49
Wheat bran, %	8.21	5.95
Corn gluten meal, %	12.13	2.19
Soybean meal, %	23.14	28.92
Soybean oil, %	2.05	2.37
L-Lysine-HCl, %	0.25	0.03
DL-Methionine, %	0.15	0.07
Limestone, %	1.37	1.41
Dicalcium phosphate, %	1.92	1.31
Salt, %	0.26	0.26
Premix ^1^, %	1.00	1.00
Total, %		
Nutrient levels ^2^		
Metabolizable energy, kcal/kg	2900	2900
Crude protein, %	22.95	19.95
Lysine, %	1.09	1.01
Methionine, %	0.49	0.39
Methionine + Cystine, %	0.91	0.73
Calcium, %	1.02	0.88
Non-phytate phosphorus, %	0.44	0.36
Se, mg/kg	<0.01	<0.01

^1^ The premix provided the following per kilogram of diets: VA 1500 IU, VD3 200 IU, VE 10 mg, VK 5.0 mg, VB12 0.01 mg, Biotin 0.15 mg, Choline chloride 1300 mg, Folic acid 0.55 mg, Pantothenic acid 10 mg, Nicotinic acid 35 mg, Fe (from ferrous sulfate), 80 mg; Cu (from copper sulfate), 8 mg; Mn (from manganese sulfate), 60 mg; Zn (from zinc sulfate), 40 mg; I (from potassium iodide), 0.35 mg. ^2^ Nutrient levels were analyzed unless otherwise noted. Crude protein, amino acids (lysine, methionine, and cystine), calcium, and total phosphorus were analyzed according to Chinese national standards GB/T 6432-2018 [28], GB/T 18246-2019 [29], GB/T 13885-2017 [30], and GB/T 6437-2018 [31], respectively. The selenium content of the basal diet was determined according to GB/T 13883-2008 [32] and was confirmed to be below 0.01 mg/kg. Metabolizable energy was calculated based on the China Feed Composition and Nutritional Value Table (31st Edition, 2020) [33], whereas non-phytate phosphorus was calculated from the analyzed total phosphorus content.

**Table 2:** Effects of selenium supplementation on body weight, average daily gain, and average daily feed intake in broilers ^1^.

Item		Body Weight			Average Daily Gain, g/d			Average Daily Feed Intake, g/d		
Group	Level, mg Se/kg	1 d, g	22 d, g	42 d, kg	1–21 d	22–42 d	1–42 d	1–21 d	22–42 d	1–42 d
CON	0	47.17	755.7	1.99	33.74	58.54	44.74	53.50	145.1	99.66
Se-Met	0.05	47.19	784.2	2.31 **	35.22	68.48	53.75 **	55.49	147.8	101.6
Se-Met	0.10	47.15	805.1	2.27 **	36.09	69.88	52.99 **	55.48	149.5	102.5
Se-Met	0.15	47.16	848.7 **	2.23 *	38.17 **	65.88	52.02 *	57.14 *	148.0	100.7
Se-Met	0.20	47.18	810.6	2.27 **	36.35	69.66	53.01 **	55.60	156.3	105.9 *
Se-Met	0.25	47.16	773.8	2.14	34.28	64.44	49.83	53.96	145.1	101.3
L-SeMC	0.05	47.15	817.4	2.33 **	36.68	72.05	54.36 **	55.01	156.9	106.5 *
L-SeMC	0.10	47.16	800.5	2.21 **	36.14	66.84	51.58 **	55.97	148.5	102.3
L-SeMC	0.15	47.17	810.6	2.23 *	36.36	67.33	51.84 *	55.43	149.5	102.4
L-SeMC	0.20	47.16	774.4	2.23 *	34.63	69.44	52.04 *	50.86	147.6	99.2
L-SeMC	0.25	47.18	780.3	2.02	34.91	60.68	46.95	52.56	134.3	93.5
Pooled SEM		0.012	6.79	0.035	0.268	2.316	0.657	0.525	3.516	1.354
*p*-Value		0.99	0.06	<0.001	0.065	0.21	<0.001	0.005	0.09	0.04
Two-way ANOVA										
Main effect										
Se type	Se-Met	47.17	803.7	2.245	36.02	67.67	52.32	55.54	149.3	102.4
	L-SeMC	47.16	797.8	2.204	35.74	67.27	51.36	53.97	147.4	100.8
Se level	0.05	47.17	802.1 ^ab^	2.318 ^ab^	35.95 ^ab^	70.26	54.06 ^a^	55.25	152.4	104.1
	0.10	47.16	805.6 ^ab^	2.243 ^ab^	36.12 ^ab^	68.36	52.28 ^ab^	55.72	149.0	102.4
	0.15	47.16	829.7 ^a^	2.228 ^ab^	37.26 ^a^	66.60	51.93 ^ab^	56.29	148.7	101.6
	0.20	47.17	792.5 ^ab^	2.253 ^a^	35.49 ^ab^	69.55	52.52 ^ab^	53.23	151.9	102.6
	0.25	47.17	773.7 ^b^	2.080 ^b^	34.59 ^b^	62.56	48.39 ^b^	53.26	139.7	97.4
*p*-Value										
Type		0.99	0.72	0.48	0.72	0.87	0.51	0.13	0.58	0.43
Level		0.89	0.02	0.01	0.02	0.06	0.02	0.06	0.15	0.17
Type × Level		0.98	0.32	0.65	0.33	0.55	0.63	0.11	0.17	0.10

^1^ Values are presented as treatment means based on six replicate pens per treatment (*n* = 6). For each trait, a single pooled standard error of the mean (pooled SEM), derived from the residual error term of the statistical model including all 11 treatment groups, is presented. Differences between each supplemented group and the control group were analyzed using one-way ANOVA followed by Dunnett’s multiple-comparisons test. Asterisks indicate significant differences from the control group: * *p* < 0.05, ** *p* < 0.01. For comparisons among all treatment groups (excluding the control), a one-way ANOVA followed by Tukey’s test was performed, and different letters (a, b) indicate significant differences (*p* < 0.05).

**Table 3:** Effects of selenium supplementation on feed conversion ratio and mortality in broilers ^1^.

Item		Feed Conversion Ratio, kg Feed/kg BW			Mortality Rate, %		
Group	Level, mg Se/kg	1–21 d	22–42 d	1–42 d	1–21 d	22–42 d	1–42 d
CON	0	1.562	2.426	2.149	8.33	12.96	20.00
Se-Met	0.05	1.545	2.167	1.950 *	1.67	15.00	16.67
Se-Met	0.10	1.540	2.151	1.938 **	5.00	12.27	16.67
Se-Met	0.15	1.497 *	2.203	1.973 *	3.33	5.00	8.33
Se-Met	0.20	1.510	2.256	2.005	5.00	10.19	15.00
Se-Met	0.25	1.537	2.267	2.013	6.67	12.22	18.33
L-SeMC	0.05	1.509	2.059 **	1.878 ***	1.67	3.33	5.00 *
L-SeMC	0.10	1.541	2.134 *	1.940 *	3.33	8.70	11.67
L-SeMC	0.15	1.500 *	2.227	1.979 *	1.67	11.85	13.33
L-SeMC	0.20	1.510	2.139 *	1.914 **	5.00	5.19	10.00
L-SeMC	0.25	1.488 *	2.238	1.960 *	3.33	8.89	11.67
Pooled SEM		0.008	0.026	0.023	1.656	2.478	2.897
*p*-Value		0.013	0.065	0.01	0.55	0.35	0.18
Two-way ANOVA							
Main effect							
Se type							
	Se-Met	1.526	2.209	1.976	4.333	10.94	15.00
	L-SeMC	1.510	2.159	1.934	3.000	7.60	10.33
Se level							
	0.05	1.527 ^ab^	2.113	1.914	1.67	9.17	10.83
	0.10	1.541 ^a^	2.142	1.939	4.17	10.49	14.17
	0.15	1.498 ^b^	2.215	1.976	2.50	8.43	10.83
	0.20	1.510 ^ab^	2.198	1.959	5.00	7.69	12.50
	0.25	1.513 ^ab^	2.253	1.986	5.00	10.56	15.00
*p*-Value							
Type		0.12	0.34	0.19	0.23	0.20	0.06
Level		0.009	0.15	0.27	0.30	0.67	0.37
Type × Level		0.30	0.68	0.61	0.67	0.23	0.20

^1^ Values are presented as treatment means based on six replicate pens per treatment (*n* = 6). For each trait, a single pooled standard error of the mean (pooled SEM), derived from the residual error term of the statistical model including all 11 treatment groups, is presented. Differences between each supplemented group and the control group were analyzed using one-way ANOVA followed by Dunnett’s multiple-comparisons test. Asterisks indicate significant differences from the control group: * *p* < 0.05, ** *p* < 0.01, and *** *p* < 0.001. For comparisons among all treatment groups (excluding the control), a one-way ANOVA followed by Tukey’s test was performed, and different letters (a, b) indicate significant differences (*p* < 0.05).

**Table 4:** Quadratic Model-Estimated Stationary Points and 95% Confidence Intervals for Selenium Supplementation ^1^.

Selenium Source	Item	Stage	Quadratic Regression Equations	R^2^	*p* Value	Stationary Point	95% Bootstrap CI
Se-Met	ADG	1–21 d	*Y* = 33.69 + 49.94*X* − 175.25*X*^2^	0.275	0.005	0.142	[0.121, 0.179]
Se-Met	ADG	22–42 d	*Y* = 60.13 + 134.94*X* − 473.40*X*^2^	0.173	0.043	0.143	[0.103, 0.225]
Se-Met	ADG	1–42 d	*Y* = 46.91 + 92.44*X* − 324.32*X*^2^	0.248	0.009	0.143	[0.144, 0.187]
Se-Met	FCR	1–21 d	*Y* = 1.59 − 1.02*X* + 3.56*X*^2^	0.193	0.029	0.143	[0.111, 0.196]
Se-Met	FCR	1–42 d	*Y* = 2.08 − 2.04*X* + 7.43*X*^2^	0.199	0.026	0.137	[0.103, 0.197]
Se-Met	ADFI	1–21 d	*Y* = 53.53 + 40.65*X* − 142.98*X*^2^	0.171	0.046	0.142	[0.110, 0.218]
Se-Met	BW	22 d	*Y* = 754.75 + 1048.53*X* − 3679.72*X*^2^	0.275	0.005	0.142	[0.121, 0.179]
Se-Met	BW	43 d	*Y* = 2.02 + 3.88*X* − 13.62*X*^2^	0.248	0.009	0.143	[0.114, 0.187]
L-SeMC	ADG	1–42 d	*Y* = 47.51 + 90.93*X* − 360.18*X*^2^	0.175	0.042	0.126	[0.078, 0.169]
L-SeMC	ADFI	1–21 d	*Y* = 54.15 + 27.74*X* − 157.07*X*^2^	0.192	0.030	0.088	[0.013, 0.134]
L-SeMC	ADFI	1–42 d	*Y* = 97.48 + 103.1*X* − 475.53*X*^2^	0.178	0.039	0.108	[0.063, 0.149]
L-SeMC	BW	43d	*Y* = 2.04 + 3.82*X* − 15.13*X*^2^	0.175	0.042	0.126	[0.078, 0.169]

^1^ Only variables with a significant quadratic term (β_2_, *p* < 0.05) are presented. Given the low R^2^ values, the estimated optima should be considered exploratory rather than definitive requirements.

**Table 5:** Effects of selenium source and level on slaughter performance of broilers ^1^.

Group	Level, mg Se/kg	Body Weight 43 d, kg	Liver Index	Spleen Index	Pectoralis Muscle Index	Gastrocnemius Muscle Index
CON	0	1.951	2.548	0.188	19.85	0.835
Se-Met	0.05	2.284 ***	2.189	0.175	19.44	0.845
Se-Met	0.10	2.267 ***	2.149	0.162	19.42	0.861
Se-Met	0.15	2.268 ***	2.252	0.114	19.89	0.807
Se-Met	0.20	2.361 ***	2.337	0.178	21.14	0.894
Se-Met	0.25	2.174 *	2.110 *	0.098 *	19.38	0.828
L-SeMC	0.05	2.333 ***	2.141	0.177	19.46	0.818
L-SeMC	0.10	2.317 ***	2.028 **	0.151	21.05	0.835
L-SeMC	0.15	2.213 **	2.271	0.116	19.32	0.857
L-SeMC	0.20	2.198 **	2.108 *	0.191	18.93	0.853
L-SeMC	0.25	2.102	2.178	0.169	19.32	0.835
Pooled SEM		0.024	0.046	0.007	0.219	0.009
*p*-Value		<0.001	0.09	0.01	0.07	0.32
Two-way ANOVA						
Main effect						
Se type	Se-Met	2.271	2.208	0.145	19.85	0.847
	L-SeMC	2.232	2.145	0.161	19.61	0.840
Se level	0.05	2.309 ^ab^	2.165	0.176 ^ab^	19.45	0.831
	0.10	2.292 ^a^	2.089	0.157 ^abc^	20.23	0.848
	0.15	2.241 ^ab^	2.261	0.115 ^c^	19.60	0.832
	0.20	2.279 ^a^	2.222	0.184 ^a^	20.04	0.874
	0.25	2.138 ^ab^	2.144	0.134 ^abc^	19.35	0.831
*p*-Value						
Type		0.28	0.46	0.16	0.46	0.57
Level		<0.001	0.23	0.045	0.54	0.32
Type × Level		0.032	0.65	0.18	0.025	0.22

^1^ Values are presented as means ± SEM based on six replicate pens per treatment (*n* = 6). For variables measured in two birds per pen, the measurements from the two birds were averaged within the pen before statistical analysis, and the pen was used as the experimental unit. A pooled SEM, calculated from the residual error of the statistical model including all 11 treatment groups, is presented for each trait. Differences from the control group were analyzed by one-way ANOVA followed by Dunnett’s multiple comparisons test. Asterisks indicate significant differences from the Control group: * *p* < 0.05, ** *p* < 0.01, *** *p* < 0.001. For comparisons among all treatment groups (excluding the control), a one-way ANOVA followed by Tukey’s test was performed, and different letters (a, b, c) indicate significant differences (*p* < 0.05).

**Table 6:** Plasma biochemical parameters of broilers fed diets with different selenium sources and levels ^1^.

Group	Level, mg Se/kg	TG mmol/L	T-CHO mmol/L	HDL mmol/L	LDL mmol/L	AST U/L	ALT U/L	T-Bil μmol/L	CRE μmol/L	UA μmol/L
CON	0	0.188	2.587	0.582	0.619	32.75	2.136	0.757	42.91	284.2
Se-Met	0.05	0.086	2.721	0.504	0.667	28.56	2.340	0.838	40.40	191.9
Se-Met	0.10	0.106	2.928	0.446	0.823	28.38	2.252	0.718	40.85	217.4
Se-Met	0.15	0.121	2.483	0.576	0.614	25.24 **	2.198	0.693	40.78	216.5
Se-Met	0.20	0.107	2.833	0.503	0.644	30.47	2.106	0.752	43.41	179.6
Se-Met	0.25	0.092	2.807	0.571	0.568	26.46	2.215	0.725	41.75	136.4 **
L-SeMC	0.05	0.082	2.598	0.735	0.644	26.16	2.661	0.703	44.76	175.7 *
L-SeMC	0.10	0.105	2.607	0.640	0.583	25.54 *	2.877	0.845	42.46	146.4 **
L-SeMC	0.15	0.138	2.763	0.776	0.790	22.45 ***	2.290	0.776	46.31 *	210.6
L-SeMC	0.20	0.157	2.549	0.581	0.662	20.60 ***	2.336	0.703	43.47	179.8
L-SeMC	0.25	0.093	2.711	0.698	0.545	20.42 ***	2.888*	0.724	44.83	154.5 *
Pooled SEM		0.026	0.086	0.121	0.022	1.238	0.166	0.038	0.547	21.79
*p*-Value		0.71	0.18	0.94	0.06	<0.001	0.049	0.58	<0.001	0.02
Two-way ANOVA										
Main effect										
Se type	Se-Met	0.102	2.754	0.520	0.663	27.82	2.222	0.745	41.44	188.4
	L-SeMC	0.115	2.646	0.686	0.645	23.03 ***	2.610 **	0.750	44.37 ***	173.4
Se level	0.05	0.084	2.660	0.620	0.655	27.36 ^a^	2.500	0.771	42.58	183.8
	0.10	0.106	2.768	0.543	0.703	26.96 ^ab^	2.564	0.782	41.66	181.9
	0.15	0.129	2.623	0.676	0.702	23.85 ^ab^	2.244	0.735	43.54	213.6
	0.20	0.132	2.691	0.542	0.653	25.54 ^ab^	2.221	0.728	43.44	179.7
	0.25	0.093	2.759	0.635	0.557	23.44 ^b^	2.552	0.724	43.29	145.4
*p*-Value										
Type		0.37	0.17	0.18	0.68	0.005	0.008	0.87	<0.001	0.51
Level		0.15	0.53	0.52	0.22	0.01	0.17	0.71	0.19	0.08
Type × Level		0.41	0.14	0.54	0.08	0.11	0.30	0.17	0.0008	0.54

^1^ Values are presented as means ± SEM based on six replicate pens per treatment (*n* = 6). For variables measured in two birds per pen, the measurements from the two birds were averaged within the pen before statistical analysis, and the pen was used as the experimental unit. A pooled SEM, calculated from the residual error of the statistical model including all 11 treatment groups, is presented for each trait. Differences from the control group were analyzed by one-way ANOVA followed by Dunnett’s multiple comparisons test. Asterisks indicate significant differences from the Control group: * *p* < 0.05, ** *p* < 0.01, *** *p* < 0.001. For comparisons among all treatment groups (excluding the control), a one-way ANOVA followed by Tukey’s test was performed, and different letters (a, b) indicate significant differences (*p* < 0.05).

**Table 7:** Selenium supplementation enhances plasma antioxidant capacity in broilers ^1^.

Group	Level, mg Se/kg	GSH-px, U/mg prot	MDA, nmol/mL	T-SOD, U/mg prot	T-AOC, U/mg prot	T-GSH, μmol/L	GSSG, μmol/g prot	GSH, μmol/g prot
CON	0	249.1	4.150	548.4	85.39	2.092	3.521	11.46
Se-Met	0.05	618.3 *	4.144	572.4	66.71	2.166	3.429	11.06
Se-Met	0.10	866.7 ***	3.755	572.7	86.19	2.184	3.648	11.33
Se-Met	0.15	1282.2 ***	3.048 **	556.8	70.74	2.196	3.293	15.25
Se-Met	0.20	2066.8 ***	3.429	552.7	78.51	2.386	3.743	7.97
Se-Met	0.25	2243.0 ***	3.107 **	518.5	65.25	2.148	2.921	9.68
L-SeMC	0.05	1395.8 ***	2.789 ***	509.5	63.61	2.285	4.201	11.61
L-SeMC	0.10	1616.4 ***	3.165 *	518.0	56.42 **	2.274	3.765	18.08 *
L-SeMC	0.15	2376.1 ***	3.410	556.5	58.79 *	2.160	3.892	14.12
L-SeMC	0.20	2211.1 ***	2.912 ***	519.5	69.54	2.021	3.630	11.15
L-SeMC	0.25	2599.7 ***	3.605	537.1	63.31	2.593	3.378	13.73
Pooled SEM		87.79	0.147	12.26	4.273	0.136	0.181	0.985
*p*-Value		<0.001	<0.001	0.04	0.003	0.83	0.14	<0.001
Two-way ANOVA								
Main effect								
Se type	Se-Met	1415.4	3.497	554.6	73.48	2.216	3.407	11.06
	L-SeMC	2039.8 ***	3.176 *	528.1 *	62.33 **	2.267	3.773*	13.74 **
Se level	0.05	1007.15 ^d^	3.467 ^a^	540.9	65.16 ^ab^	2.226	3.815	11.34 ^ab^
	0.10	1241.35 ^c^	3.460 ^a^	545.4	71.30 ^ab^	2.229	3.706	14.71 ^a^
	0.15	1829.0 ^b^	3.229 ^ab^	556.6	64.77 ^b^	2.178	3.592	14.68 ^a^
	0.20	2139.0 ^ab^	3.170 ^b^	536.1	74.02 ^a^	2.203	3.686	9.56 ^b^
	0.25	2421.5 ^a^	3.356 ^ab^	527.8	64.28 ^b^	2.371	3.150	11.70 ^ab^
*p*-Value								
Type		<0.001	0.01	0.02	0.008	0.80	0.043	0.048
Level		<0.001	0.02	0.56	0.017	0.67	0.42	0.005
Type × Level		0.001	<0.001	0.07	0.17	0.21	0.14	0.07

^1^ Values are presented as means ± SEM based on six replicate pens per treatment (*n* = 6). Each sample was analyzed in technical triplicate, and the technical replicates were averaged before statistical analysis. For variables measured in two birds per pen, the two bird-level measurements were then averaged within the pen, and the pen was used as the experimental unit. A pooled SEM, calculated from the residual error of the statistical model including all 11 treatment groups, is presented for each trait. Differences from the control group were analyzed by one-way ANOVA followed by Dunnett’s multiple comparisons test. Asterisks indicate significant differences from the Control group: * *p* < 0.05, ** *p* < 0.01, *** *p* < 0.001. For comparisons among all treatment groups (excluding the control), a one-way ANOVA followed by Tukey’s test was performed, and different letters (a–d) indicate significant differences (*p* < 0.05).

**Table 8:** Meat quality of broilers as influenced by selenium source and supplementation level ^1^.

Item		Shear Force, N	Drip Loss, %	Lightness (L*)		Redness (a*)		Yellowness (b*)		pH	
Group	Level, mg Se/kg			45 min	24 h	45 min	24 h	45 min	24 h	45 min	24 h
CON	0	32.60	5.17	49.14	57.50	10.18	8.74	7.58	9.72	6.04	5.32
Se-Met	0.05	34.95	5.10	47.44	54.47 *	10.94	9.57	7.80	11.16	5.93	5.29
Se-Met	0.10	48.51	4.64	48.16	56.39	10.10	8.99	7.17	9.82	6.02	5.38
Se-Met	0.15	32.86	4.48	46.95	52.44 ***	10.38	10.05 *	7.15	8.83	5.95	5.48 ***
Se-Met	0.20	32.14	4.46	47.27	52.02 ***	10.78	10.07 *	8.76	10.38	6.04	5.46 **
Se-Met	0.25	35.11	4.37	45.96 **	51.68 ***	10.95	10.13 *	8.16	10.56	5.96	5.47 **
L-SeMC	0.05	39.17	4.55	48.63	52.75 ***	9.67	9.34	9.15	11.43 *	6.19	5.43 *
L-SeMC	0.10	33.67	4.73	45.58 ***	50.43 ***	11.13	10.43 **	8.01	9.45	6.17	5.50 ***
L-SeMC	0.15	28.58	4.43	47.25	53.40 **	10.38	9.66	8.61	10.69	6.01	5.39
L-SeMC	0.20	34.62	5.07	47.70	52.06 ***	10.34	9.51	9.09	11.07	5.94	5.45 **
L-SeMC	0.25	36.33	6.28	47.22	52.92 ***	10.97	10.44 **	6.92	8.78	6.02	5.44 *
Pooled SEM		6.238	0.316	0.487	0.623	0.435	0.138	0.512	0.187	0.043	0.011
*p*-Value		0.16	0.34	0.003	<0.001	0.77	0.001	0.51	<0.001	0.25	<0.001
Two-way ANOVA											
Main effect											
Se type	Se-Met	36.71	4.61	47.16	53.40	10.63	9.76	7.81	10.15	5.98	5.41
	L-SeMC	34.47	5.01	47.27	52.31 *	10.50	9.88	8.36	10.28	6.06	5.44
Se level	0.05	37.06	4.82	48.04	53.61	10.31	9.46	8.48	11.29 ^a^	6.06	5.36 ^b^
	0.10	41.09	4.68	46.87	53.41	10.61	9.71	7.59	9.63 ^b^	6.09	5.44 ^ab^
	0.15	30.72	4.45	47.1	52.92	10.38	9.85	7.88	9.76 ^b^	5.98	5.44 ^ab^
	0.20	33.38	4.76	47.49	52.04	10.56	9.79	8.93	10.73 ^ab^	5.99	5.45 ^a^
	0.25	35.72	5.33	46.59	52.30	10.96	10.29	7.54	9.67 ^b^	5.99	5.45 ^a^
*p*-Value											
Type		0.41	0.29	0.64	0.02	0.65	0.42	0.33	0.46	0.09	0.24
Level		0.31	0.45	0.13	0.08	0.78	0.09	0.47	<0.001	0.56	0.01
Type × Level		0.20	0.28	0.03	<0.001	0.15	0.006	0.43	<0.001	0.16	<0.001

^1^ Values are presented as means ± SEM based on six replicate pens per treatment (*n* = 6). Repeated measurements within each meat sample were averaged to obtain one value per bird. The measurements from the two birds sampled from the same pen were then averaged within the pen before statistical analysis, and the pen was used as the experimental unit. A pooled SEM, calculated from the residual error of the statistical model including all 11 treatment groups, is presented for each trait. Differences from the control group were analyzed by one-way ANOVA followed by Dunnett’s multiple comparisons test. Asterisks indicate significant differences from the Control group: * *p* < 0.05, ** *p* < 0.01, *** *p* < 0.001. For comparisons among all treatment groups (excluding the control), a one-way ANOVA followed by Tukey’s test was performed, and different letters (a, b) indicate significant differences (*p* < 0.05).

**Table 9:** Model-estimated relative potency of L-SeMC compared with Se-Met for selected measured outcomes in white-feathered broilers ^1^.

Item	Equation	R^2^	Model-Estimated Relative Potency Ratio (L-SeMC:Se-Met)	95% CI
BW_43d	*y* = 1.9900 + 0.2290 [1 − e^(−502912*x*1−931358*x*2)^]	0.622	1.85	1.65–2.35
GSH-Px	*y* = 214.9 + 2656.1 [1 − e^(−4.5583*x*1−8.7941*x*2)^]	0.947	1.93	1.52–2.21

^1^ The model was fitted using 66 pen-level observations, comprising six replicate pens within each of the 11 dietary treatments. The relative-potency ratio was calculated as b_2_/b_1_, with Se-Met assigned a reference value of 1.00. The 95% confidence intervals were obtained by nonparametric bootstrap resampling at the pen level, with the nonlinear model refitted for each bootstrap sample.

## Data Availability

The original contributions presented in the study are included in the article/Supplementary Materials, further inquiries can be directed to the corresponding authors.

## Supplementary Materials

The following supporting information can be downloaded at https://www.mdpi.com/article/10.3390/ani16182882/s1, Table S1: Analyzed selenium concentrations in the experimental diets; Table S2: Significance of quadratic regression trends for growth performance, plasma biochemical, and plasma antioxidant parameters.

## Abbreviations

The following abbreviations are used in this manuscript:

Se	Selenium
Se-Met	selenomethionine
L-SeMC	L-selenomethylselenocysteine
GSH-Px	glutathione peroxidase
ADG	average daily gain
FCR	feed conversion ratio
MDA	malondialdehyde
IACUC	Institutional Animal Care and Use Committee
ND-IB	Newcastle disease–infectious bronchitis
IBD	infectious bursal disease
CON	control group
BW_22d	average body weight per replicate at 22 d
BW_42d	average body weight per replicate at 42 d
ADFI	average daily feed intake
TG	triglycerides
T-CHO	total cholesterol
HDL	high-density lipoprotein cholesterol
LDL	low-density lipoprotein cholesterol
AST	aspartate aminotransferase
ALT	alanine aminotransferase
T-Bil	total bilirubin
CRE	creatinine
UA	uric acid
T-SOD	total superoxide dismutase
T-AOC	total antioxidant capacity
T-GSH	total glutathione
GSSG	oxidized glutathione
GSH	glutathione
N	shear force
L*	lightness
a*	redness
b*	yellowness
*X*_opt_	optimal concentration
r_s_	correlation coefficient
