# Supplementary material for: L-Selenomethylselenocysteine Improves Growth Performance, Antioxidant Status, and Meat Quality in White-Feathered Broilers More Effectively than Selenomethionine
Source: Animals (Basel). 2026 Sep 13;16(18):2882. doi: 10.3390/ani16182882 (PMC13603490; doi:10.3390/ani16182882)
Supplement: Supplementary file 1 [file animals-16-02882-s001.zip › Table S1.pdf]

Table S1 Analyzed selenium concentrations in the experimental diets.

	Analyzed selenium concentration (mg/Kg)	
Supplemental Se level (mg Se/Kg)	Se-Met	L-SeMC
0 (CON)	<0.01	<0.01
0.05	0.124 ± 0.02	0.115 ± 0.03
0.10	0.248 ± 0.03	0.231 ± 0.02
0.15	0.373 ± 0.03	0.346 ± 0.02
0.20	0.500 ± 0.02	0.461 ± 0.03
0.25	0.621 ± 0.01	0.577 ± 0.04

Values are means of triplicate analyses. Selenium concentrations in the experimental diets were determined by atomic fluorescence spectrometry according to GB/T 5009.93-2017. The selenium concentration in the basal diet was below the detection limit (0.01 mg/kg).
