# Supplementary material for: L-Selenomethylselenocysteine Improves Growth Performance, Antioxidant Status, and Meat Quality in White-Feathered Broilers More Effectively than Selenomethionine
Source: Animals (Basel). 2026 Sep 13;16(18):2882. doi: 10.3390/ani16182882 (PMC13603490; doi:10.3390/ani16182882)
Supplement: Supplementary file 1 [file animals-16-02882-s001.zip › Table S2.pdf]

Supplementary Table S2 Significance of quadratic regression trends for growth performance, plasma biochemical, and plasma antioxidant parameters.

	Quadratic regression analysis	
Item	Se-Met	L-SeMC
Body Weight		
1d, g	NS	NS
22d, g	Q**	NS
43d, kg	Q**	Q*
Average daily gain, g/d		
1~21d	Q**	NS
22~42d	Q*	NS
1~42d	Q**	Q*
Average daily feed intake, g/d		
1~21d	Q*	Q*
22~42d	NS	NS
1~42d	NS	Q*
Feed conversion ratio		
1~21d	Q*	Q*
22~42d	NS	NS
1~42d	Q*	NS
Mortality rate, %		
1~21d	NS	NS
22~42d	NS	NS
1~42d	NS	NS
TG, mmol/L	NS	NS
T-CHO, mmol/L	NS	NS
HDL-C, mmol/L	NS	NS
LDL-C, mmol/L	NS	NS
AST, U/L	NS	NS
ALT, U/L	NS	NS
CRE, umol/L	NS	NS
UA, umol/L	Q***	Q**
GSH-px, U/mg prot	Q***	Q***
GSH, μmol/g prot	NS	NS
GSSG, μmol/g prot	NS	NS
T-AOC, U/mg prot	NS	NS
T-SOD, U/mg prot	NS	NS
MDA, nmol/mL	Q***	Q***

NS indicates no significant quadratic trend. Q indicates a significant quadratic regression trend. Q*, Q**, and Q*** indicate significant quadratic regression trends at $P < 0.05$, $P < 0.01$, and $P < 0.001$, respectively.
